# Supplementary material for: ASO targeting RBM3 temperature‐controlled poison exon splicing prevents neurodegeneration in vivo
Source: EMBO Mol Med. 2023 Mar 22;15(5):e17157. doi: 10.15252/emmm.202217157 (PMC10165353; doi:10.15252/emmm.202217157)
Supplement: Supplementary file 1 — Expanded View Figures PDF [file EMMM-15-e17157-s009.pdf]

Expanded View Figures

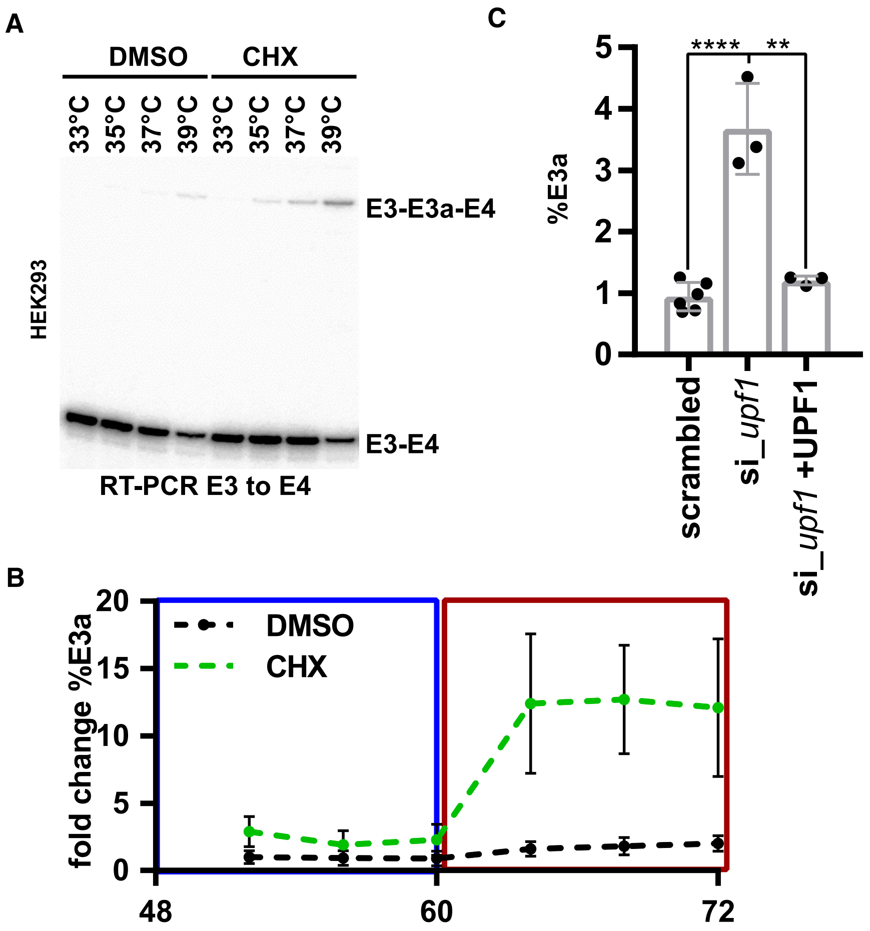

Figure EV1. RBM3 intron 3 contains an evolutionary conserved heat-induced poison exon.

A Representative gel image showing heat-induced and CHX-stabilized formation of the E3a isoform in human HEK293 cells (quantification in Fig 1C).

B Rhythmic RBM3 E3a regulation. HEK293 cells were pre-entrained with square-wave temperature cycles (12 h 34°C/12 h 38°C) for 48 h. For the last 24 h, cells were treated with DMSO or CHX every 4 h and harvested after 4 h and analyzed by splicing-sensitive RT–PCR ($n = 6$, mean ± s.d.).

C RBM3 E3a stabilization in response to UPF1 knockdown and rescue (mean ± s.d., $n = 3$, for scrambled $n = 6$, all individual data points are shown) (Colombo *et al*, 2017). Statistical significance was determined by unpaired *t*-test and is indicated by asterisks: *P*-values: $^{**}P < 0.01$, $^{****}P < 0.0001$.

© 2023 The Authors

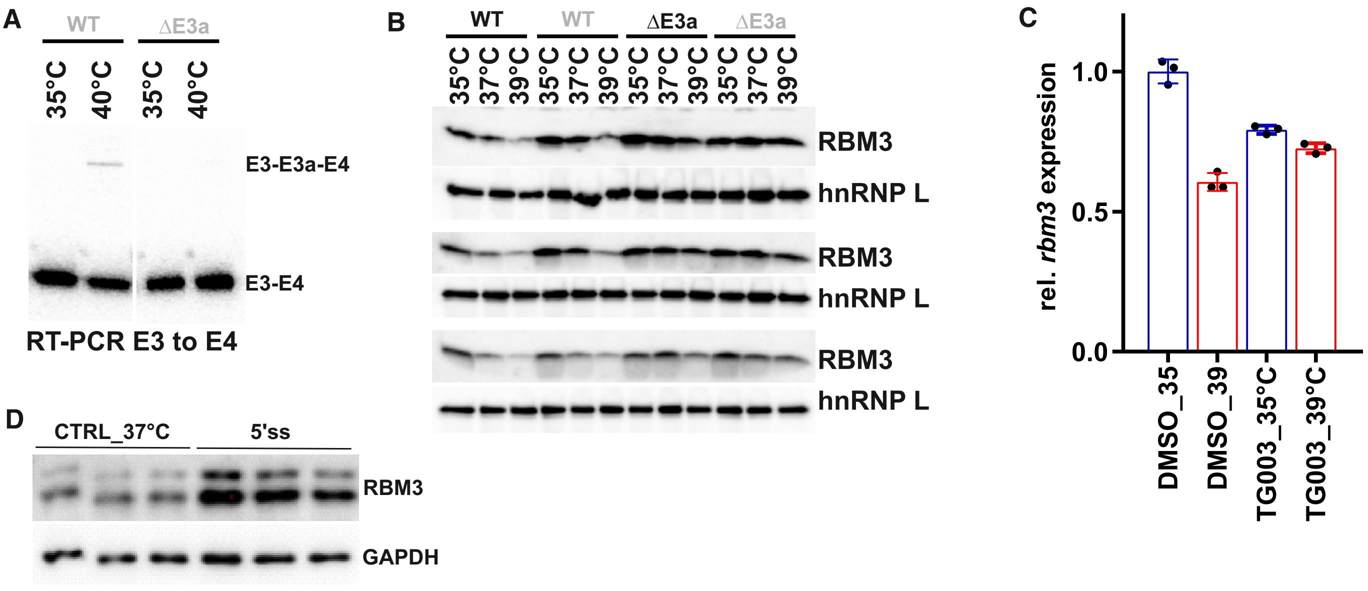

Figure EV2. E3a controls temperature-dependent RBM3 expression.

A An RT–PCR in CHX-treated HEK293 cells confirms CRISPR/CAS9-mediated removal of E3a in a DE3a clone at the RNA level.

B Western blot analysis of RBM3 protein expression, hnRNP L served as a loading control. Two independent WT and ΔE3a clones are shown in biological triplicates. Quantification in Fig 2C.

C Inhibition of CLK1/4 kinase by TG003 abolishes the effect of temperature on *rbm3* expression. Whippet-derived TpM values are shown relative to DMSO 35°C (mean ± s.d., $n = 3$, all individual data points are shown). This reveals an almost twofold difference in *rbm3* levels comparing 6 h DMSO 35 vs. 39°C. Note that this is basically abolished by adding TG003 during the shift from 39 to 35°C (Haltenhof *et al*, 2020).

D Blocking rbm3 E3a inclusion induces RBM3 protein levels. Primary hippocampal neurons were transfected with the indicated MOs for 48 h and investigated by Western blotting. GAPDH served as a loading control. Quantification in Fig 2E.

 © 2023 The Authors

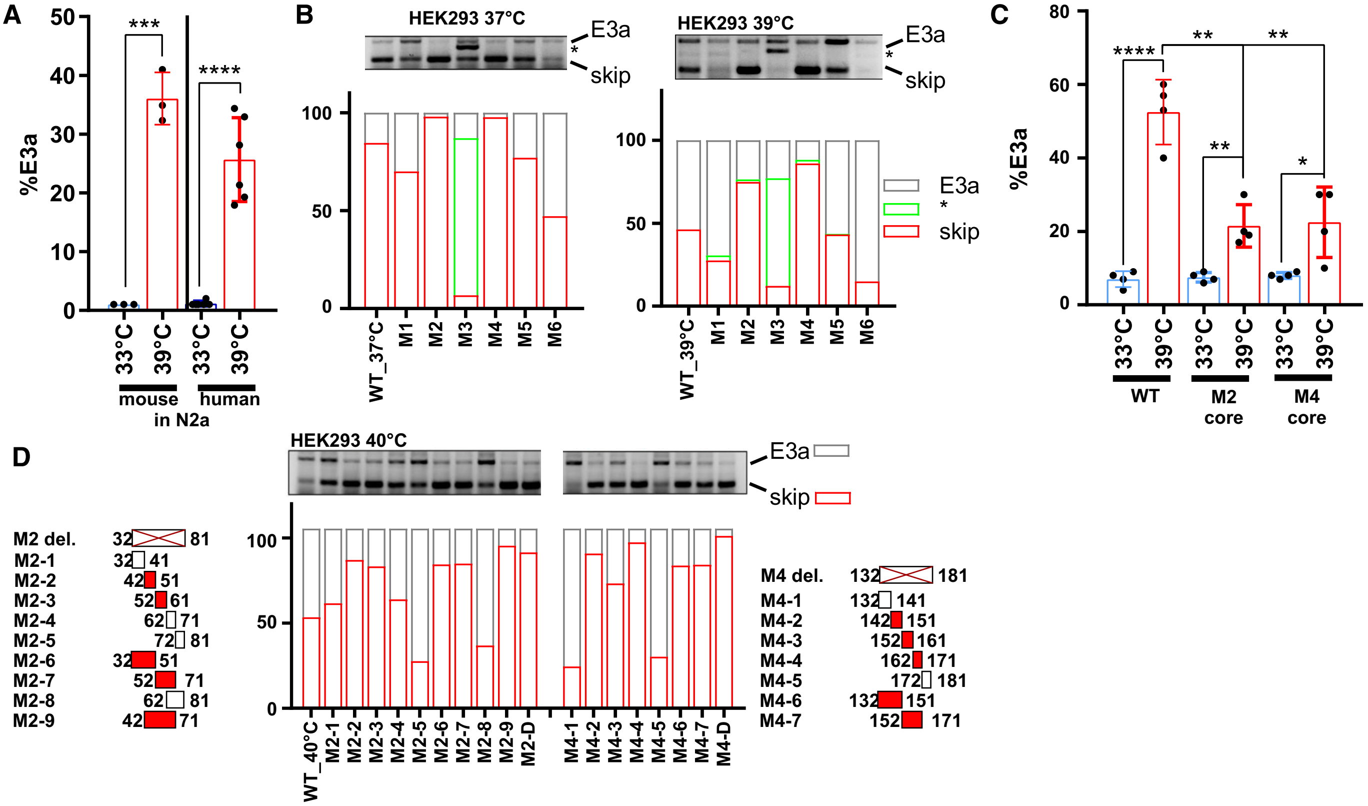

Figure EV3. Mapping cis-regulatory elements controlling RBM3 E3a inclusion.

A An RBM3 minigene reproduces temperature-controlled E3a inclusion. Human and mouse minigenes were transfected into N2a cells and analyzed as in Fig 3A (mean ± s.d., $n = 3$–6, all individual data points are shown).

B Systematic mutational screening for regulatory elements (see Table EV1). The indicated sequences were replaced by sequences from human beta-globin. M2, M4, and M5 contain the same exon 2 sequence from beta-globin exon 2. Only in the M2 and M4 context these sequences prevent inclusion, ruling out the possibility that we included a silencer element. M1 and M3 contain sequences of the beta-globin 3'ss, M6 the beta-globin 5'ss (these mutations do not result in an increased splice site strength). E3a inclusion in HEK293 at 37 and 39°C was investigated by minigene-specific RT–PCR. On top, a representative gel is shown. Below, quantifications of the detected isoforms (mean, $n = 2$). Note that replacing the internal 3'ss with a globin 3'ss promotes its usage.

C Temperature response of the indicated minigenes. We deleted the evolutionary conserved core of the M2 and M4 regions. Briefly, M2-2 and M2-3 are 100% conserved between human and mouse. Thus, M2-2 and M2-3 are regarded as core sequence of the M2 enhancer to be deleted in hRBM3 minigene. For the M4 region, M4-3 is the central region of the conserved sequence. Therefore, M4-3 and a part of the upstream sequence of M4-4 were deleted as core sequence of M4 mutant in the hRBM3 minigene (mean ± s.d., $n = 4$, all individual data points are shown).

D Detailed mutational screening of the M2 region (borders indicated on the left) and M4 region (borders indicated on the right). See Table EV1 for sequences. In M2 or M4 del. The M2 or M4 sequences are removed (and not replaced). In M2-1 to M2-9 and M4-1 to M4-7, the indicated sequences are replaced by human beta-globin exon 2 sequences from the same relative position. On top, a representative PCR image is shown. Below, quantifications of the detected isoforms (mean, $n = 2$). In all panels, statistical significance was determined by unpaired, two-sided t-test and is indicated by asterisks: P-values: $^*P < 0.05$, $^{**}P < 0.01$, $^{***}P < 0.001$, $^{****}P < 0.0001$.

© 2023 The Authors

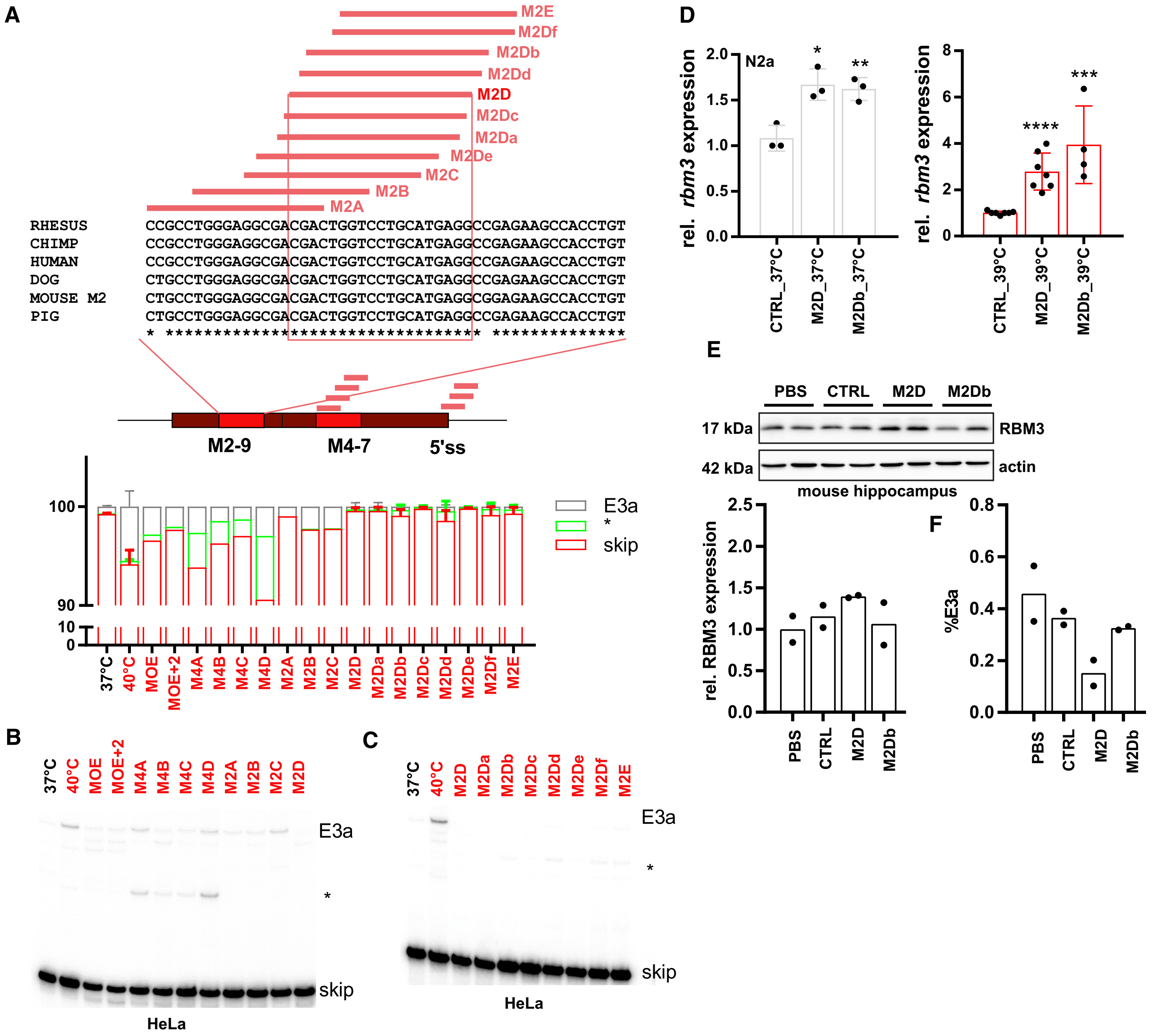

Figure EV4. Screening for ASO sequences that mediate RBM3 E3a exclusion.

A–C ASOs targeting M2-9, M4-7, or the 5'ss (see Table EV2) prevent RBM3 E3a inclusion in human HeLa cells. In (A), ASO-binding sites within the M2, M4, and the 5'ss region are indicated in the middle. On top, for the mouse M2 region an alignment of mouse, human, chimp, rhesus, dog, and pig sequences is shown. ASO-binding sites are indicated, and the 100% conserved binding site of M2D is boxed. ASOs were transfected and cells were kept at 40°C for 24 h. Control samples at 37 and 40°C are shown. All samples were treated with CHX for the last 4 h. Exon 3a inclusion was investigated by splicing-sensitive radioactive RT–PCR. In (A) (bottom), a quantification is shown (mean ± s.d., $n = 2$; $n = 5$ for all M2D variants and M2E, $n = 1$ for M4B). In (B) and (C) representative gels are shown. The asterisk marks the use of internal 5′ and 3'ss that is promoted by all ASOs targeting the M4 region. Note that all variants targeting the M2D region almost quantitatively prevent E3a inclusion and, at 40°C, lead to inclusion levels that are lower than the one observed at 37°C for control cells.

D M2D and M2Db induce *rbm3* mRNA expression in mouse N2a cells. ASOs were transfected for 24 h at 37°C (left) or at 39°C (right). rbm3 induction was measured relative to a CTRL ASO and relative to HPRT expression (mean ± s.d., $n \geq 3$, all individual data points are shown; unpaired *t*-test derived *P*-value $^*P < 0.5$, $^{**}P < 0.01$, $^{***}P < 0.001$, $^{****}P < 0.0001$).

E M2D, but not M2Db, induces RBM3 protein expression *in vivo* (100 μg dose per mouse). Hippocampus samples from two independent mice per condition were analyzed by Western blotting (top), and RBM3 signal was quantified relative to Actin and PBS (bottom, mean ± s.d., $n = 2$).

F M2D, but not M2Db, reduces E3a inclusion *in vivo* (300 μg dose per mouse). Cerebellum RNA samples from two independent mice per condition were analyzed by splicing-sensitive RT–PCR, and %E3a signal was quantified (mean ± s.d., $n = 2$).

© 2023 The Authors
