# Supplementary material for: ASO targeting RBM3 temperature‐controlled poison exon splicing prevents neurodegeneration in vivo
Source: EMBO Mol Med. 2023 Mar 22;15(5):e17157. doi: 10.15252/emmm.202217157 (PMC10165353; doi:10.15252/emmm.202217157)
Supplement: Supplementary file 4 — PDF+ [file EMMM-15-e17157-s003.pdf]

Report

SOURCE DATA · TRANSPARENT PROCESS · OPEN ACCESS

EMBO Molecular Medicine

ASO targeting RBM3 temperature-controlled poison exon splicing prevents neurodegeneration *in vivo*

Marco Preußner[1,*,†], Heather L Smith[2,3,†], Daniel Hughes[2,‡], Min Zhang[1,‡], Ann-Kathrin Emmerichs[1], Silvia Scalzitti[1], Diego Peretti[2], Dean Swinden[2,3], Alexander Neumann[1,4], Tom Haltenhof[1,4], Giovanna R Mallucci[2,3,**] & Florian Heyd[1,***]

Abstract

Neurodegenerative diseases are increasingly prevalent in the aging population, yet no disease-modifying treatments are currently available. Increasing the expression of the cold-shock protein RBM3 through therapeutic hypothermia is remarkably neuroprotective. However, systemic cooling poses a health risk, strongly limiting its clinical application. Selective upregulation of RBM3 at normothermia thus holds immense therapeutic potential. Here we identify a poison exon within the RBM3 gene that is solely responsible for its cold-induced expression. Genetic removal or antisense oligonucleotide (ASO)-mediated manipulation of this exon yields high RBM3 levels independent of cooling. Notably, a single administration of ASO to exclude the poison exon, using FDA-approved chemistry, results in long-lasting increased RBM3 expression in mouse brains. In prion-diseased mice, this treatment leads to remarkable neuroprotection, with prevention of neuronal loss and spongiosis despite high levels of disease-associated prion protein. Our promising results in mice support the possibility that RBM3-inducing ASOs might also deliver neuroprotection in humans in conditions ranging from acute brain injury to Alzheimer's disease.

Keywords alternative splicing coupled to nonsense-mediated decay; hypothermia; neurodegenerative diseases; neuroprotection; RBM3
Subject Categories Genetics, Gene Therapy & Genetic Disease; Molecular Biology of Disease; Neuroscience
DOI 10.15252/emmm.202217157 | Received 9 November 2022 | Revised 16 February 2023 | Accepted 18 February 2023 | Published online 22 March 2023
EMBO Mol Med (2023) 15: e17157

Introduction

The expression of glycine-rich RNA-binding proteins upon cooling, such as CIRBP (Cold-induced RNA-binding protein) and RBM3 (RNA-binding motif-3), was originally described in the 1990s (Danno *et al*, 1997; Nishiyama *et al*, 1997). However, despite evolutionary conservation (Ciuzan *et al*, 2015) and extreme temperature sensitivity of this phenomenon (Jackson *et al*, 2015; Los *et al*, 2022), the mechanistic basis for cold-induced RBM3 expression has remained enigmatic. Our recent identification of temperature-regulated alternative splicing coupled to nonsense-mediated decay (NMD) provides a global mechanism for the control of temperature-dependent gene expression (Neumann *et al*, 2020). The NMD pathway recognizes mRNA isoforms containing premature termination codons (PTCs) and targets these mRNAs for degradation, thus allowing splicing-controlled regulation of gene expression (Lykke-Andersen & Jensen, 2015). NMD-inducing poison isoforms are frequently found in RNA-binding proteins (Neumann *et al*, 2020) and exclusion of a poison exon upon cooling in CIRBP provides an explanation for cold-induced expression (Haltenhof *et al*, 2020). While CIRBP has diverse functions ranging from circadian sleep homeostasis to inflammation and cancer (Morf *et al*, 2012; Qiang *et al*, 2013; Lujan *et al*, 2018; Hoekstra *et al*, 2019), the closely related RBM3 protein is strongly associated with the neuroprotective effect of hypothermia. This has been observed in scenarios ranging from *in vitro* protection from forced apoptosis of neuronal cell lines and brain slices (Chip *et al*, 2011) to profound neuroprotective effects *in vivo*, where RBM3 induction by cooling or over-expression restores memory, prevents synapse and neuronal loss, and extends survival in preclinical mouse models of prion and Alzheimer's disease (Peretti *et al*, 2015, 2021). More recently, RBM3 has been shown to stimulate neurogenesis in rodent brain after hypoxic–ischemic brain injury (Zhu *et al*, 2019) and to protect against neurotoxin effects in neuronal cell lines (Yang *et al*, 2019). Therapeutic hypothermia is used in some

1 Institut für Chemie und Biochemie, RNA Biochemie, Freie Universität Berlin, Berlin, Germany
2 UK Dementia Research Institute and Department of Clinical Neurosciences, University of Cambridge, Cambridge, UK
3 Altos Labs, Cambridge Institute of Science, Cambridge, UK
4 Omiqa Bioinformatics, Berlin, Germany
*Corresponding author. Tel: +49 30 838 70703; E-mail: mpreussner@zedat.fu-berlin.de
**Corresponding author. Tel: +44 7766 924461; E-mail: gmallucci@altoslabs.com
***Corresponding author. Tel: +49 30 838 62938; E-mail: florian.heyd@fu-berlin.de
†These authors contributed equally to this work
‡These authors contributed equally to this work

© 2023 The Authors. Published under the terms of the CC BY 4.0 license.

clinical settings for neuroprotection, including neonatal hypoxic-ischemic encephalopathy, head injury (Shankaran *et al*, 2005; Shankaran, 2012; Azzopardi *et al*, 2014; Thayyil *et al*, 2021), stroke, and during cardiac surgery in adults (Hypothermia after Cardiac Arrest Study Group, 2002; Nielsen *et al*, 2013; Bernard *et al*, 2016; Lascarrou *et al*, 2019; Dankiewicz *et al*, 2021). While the mechanisms of hypothermia-induced neuroprotection in humans are not fully understood, RBM3 is used as a biomarker of success in intensive care unit (ICU) patients exposed to hypothermia (Rosenthal *et al*, 2019). Induced cooling in humans requires an ICU setup and is not without risk, with high prevalence of blood clots, pneumonia, and other complications, strongly limiting its clinical use. Inducing RBM3 without cooling would bypass these risks and requirements and could represent a much-needed new neuroprotective strategy broadly applicable in conditions ranging from stroke and brain injury to Alzheimer's and other neurodegenerative diseases. Therefore, a detailed mechanistic understanding of cold-induced RBM3 expression offers immense therapeutic potential.

Results and Discussion

We hypothesized that cold-induced RBM3 expression, similar to CIRBP expression (Haltenhof *et al*, 2020), could be regulated via cold-induced exclusion of a poison isoform that is present at normal or high temperatures. While a global splicing analysis in primary mouse hepatocytes did not reveal temperature-controlled alternative splicing in *Rbm3* (Neumann *et al*, 2020), a more focused analysis revealed an uncharacterized exon containing seven PTCs within the evolutionarily conserved intron 3. This exon, which we call exon 3a (E3a), is not included in the mRNA at colder temperature (34°C), but E3a-containing isoforms are detectable at warm temperature (38°C) and become strongly stabilized upon NMD inhibition via cycloheximide (CHX) (Fig 1A). Using splicing-sensitive radioactive RT–PCRs, we confirmed a very similar temperature-controlled E3a splicing pattern in mouse primary hippocampal neurons (Fig 1B). Consistent with high evolutionary conservation (Fig 1A, bottom) and an almost identical E3a sequence in humans, we also find temperature-controlled inclusion of the human *RBM3* E3a homolog. In HEK293 cells, E3a inclusion is highly temperature responsive within the physiologically relevant temperature range between 33 and 39°C, correlating with highly temperature-sensitive *RBM3* expression (Figs 1C and D and EV1A). In addition, *RBM3* E3a inclusion responds quickly, within 4 h, to an external square wave temperature rhythm (Fig EV1B) and could therefore control *RBM3* expression in response to circadian body temperature changes (Morf *et al*, 2012; Liu *et al*, 2013). The presence of seven PTCs within E3a (Fig 1A) and the strong stabilization upon addition of the translation inhibitor CHX suggest NMD-mediated degradation of the E3a isoform. To provide further evidence for degradation via the NMD pathway, we investigated stabilization of the E3a-containing isoform in response to NMD factor knockdowns (Colombo *et al*, 2017). In human cells, the *RBM3* E3a isoform is reversibly stabilized upon *UPF1* knockdown and dramatically and reversibly stabilized (~ 75% NMD isoform) upon *SMG6/7* double knockdown (Figs 1E and EV1C). In summary, these data identify an uncharacterized poison exon in *RBM3* that could control cold-induced *RBM3* expression through cooling-induced skipping and evasion of NMD.

To address an involvement of E3a in temperature-controlled *RBM3* expression, we used CRISPR/Cas9-mediated genome editing to generate cell lines lacking *RBM3* E3a. After clonal selection, we obtained two homozygous cell lines, derived from distinct guide RNA pairs (Figs 2A and EV2A). While two control cell lines, generated from px459 empty vector-transfected cells, showed strong temperature-controlled *RBM3* mRNA and protein expression, this was abrogated in cell lines lacking E3a (Figs 2B and C and EV2B). Importantly, cell lines lacking E3a did not only lose *RBM3* temperature sensitivity but showed a constantly high *RBM3* expression level, that in control cells was only reached at low temperature. These data suggest that temperature-controlled alternative splicing coupled to NMD is the main mechanism that controls *RBM3* expression levels in the physiologically relevant temperature range. Consistent with this, recent work has implicated several splicing factors, among others HNRNPH1, in regulating *RBM3* expression through poison exon splicing (Lin *et al*, 2022). Temperature-dependent phosphorylation of SR proteins likely contributes to this regulation (Preussner *et al*, 2017; Haltenhof *et al*, 2020), as the effect of temperature on *RBM3* expression is strongly reduced in conditions with inhibited CDC-like kinases (CLKs; Fig EV2C). Importantly, this mechanism offers the possibility to manipulate RBM3 expression by modulating alternative splicing of E3a using antisense oligonucleotides (ASOs). A splice-modulating ASO is already in clinical use to treat spinal muscular atrophy (SMA) in humans (Finkel *et al*, 2016) thus representing an established approach to therapeutically manipulate alternative splicing in the human nervous system with beneficial effect. As proof of principle, we used a splice-site blocking morpholino (MO) directed against the 5'ss of *RBM3* E3a. This MO induced *RBM3* mRNA levels substantially in HEK293 cells, confirming that *RBM3* expression can be controlled in *trans* by targeting splicing of E3a (Fig 2D; see also below). Importantly, MO transfection also induced RBM3 protein expression in mouse primary hippocampal neurons at 37°C (Figs 2E and EV2D), validating our findings in a setup relevant for neuroprotection/degeneration. Together, these data provide strong evidence for splicing-controlled *RBM3* expression and identify E3a as a potential therapeutic target for a neuroprotective increase of RBM3 expression at normothermia.

Manipulation of (non-productive) alternative splicing with ASOs has broad therapeutic potential (Bennett *et al*, 2019; Lim *et al*, 2020), but the efficiency of induced exon skipping is strongly dependent on the exact sequence and chemistry of the oligonucleotide (Erdos *et al*, 2021; Puttaraju *et al*, 2021). Therefore, the design of the most potent ASOs often requires intensive screening. To narrow down potential target sites for antisense-based therapeutics, we started with a minigene analysis. In a minigene context, NMD isoforms are not degraded as the minigene-derived RNA is not translated, and minigenes allow systematic mutagenesis to decipher *cis*-regulatory elements. We cloned the human or mouse genomic sequence comprising *RBM3* exons 3 to 4 and analyzed minigene splicing after transfection of (human) HEK293 (Figs 3A and B and EV3A) and (mouse) neuroblastoma N2a cells, respectively (Fig EV3A). Similar to endogenous splicing, minigene splicing responds to temperature in a gradual manner (Fig 3B) and is indistinguishable between mouse and human minigenes (Fig EV3A), indicating that the minigene contains all *cis*-regulatory elements required for evolutionarily conserved temperature-controlled alternative splicing. Screening mutagenesis by replacing 50 nucleotide windows with human *beta*-

 © 2023 The Authors

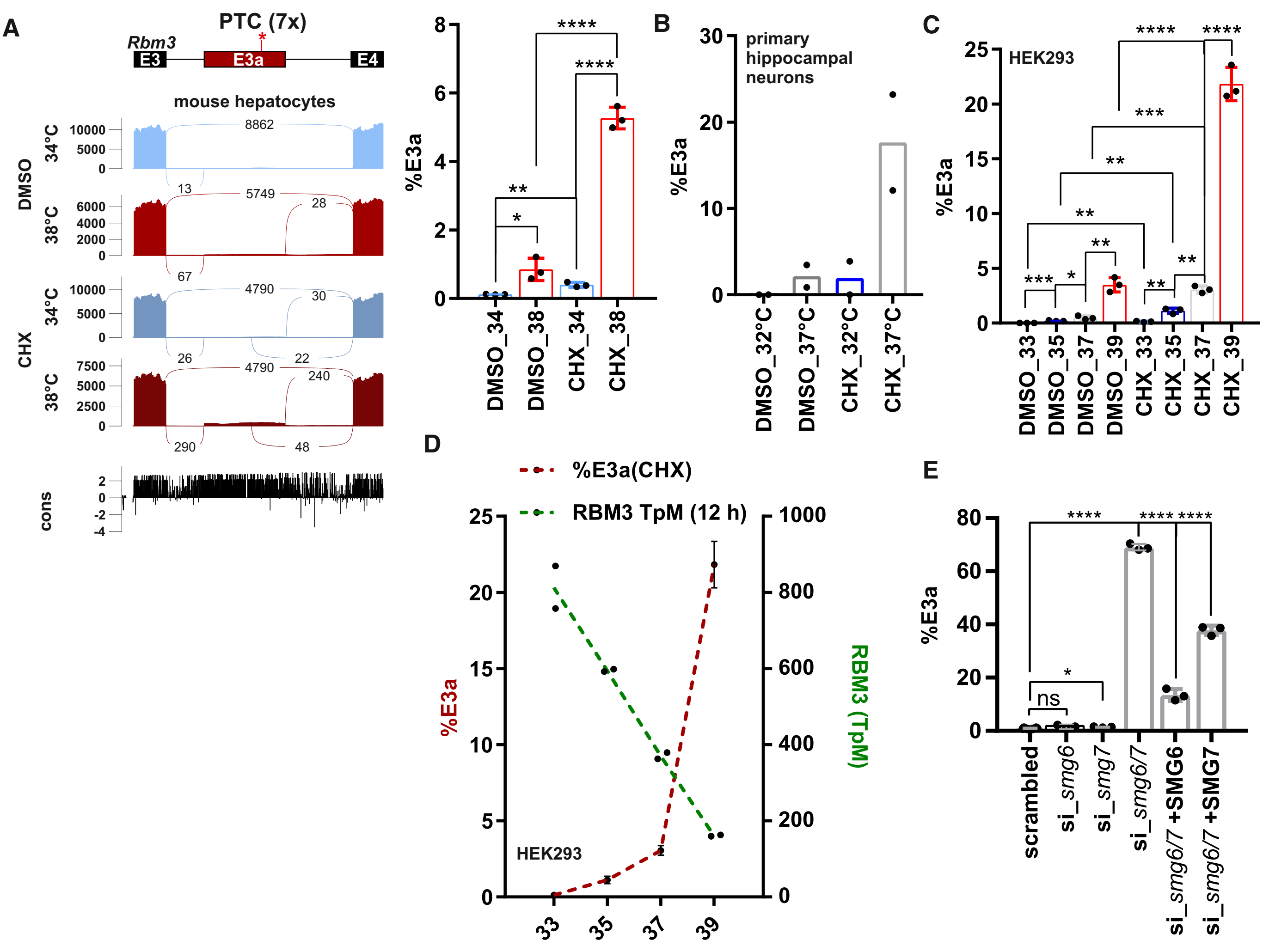

Figure 1. *RBM3* intron 3 contains an evolutionary conserved heat-induced poison exon.

A Sashimi blot identifies an uncharacterized exon (E3a; with 7 premature termination codons: PTC) within *Rbm3* intron 3. Mouse primary hepatocytes were incubated at 34 or 38°C with or without the translation inhibitor cycloheximide (CHX, DMSO as solvent control) and analyzed by RNA sequencing. Below the simplified exon-intron structure, the Sashimi plot shows the distribution of raw sequencing reads. Exon–Exon junction reads are indicated by the numbers connecting the exons. At the bottom, high sequence conservation across placental species is indicated. Quantification of %E3a inclusion in RNA sequencing samples is shown on the right (mean ± s.d., $n = 3$, all individual data points are shown) (Neumann *et al*, 2020).

B Quantification of radioactive splicing-sensitive RT–PCRs confirm heat-induced and CHX-stabilized formation of the E3a-containing isoform at warmer temperatures in primary hippocampal neurons (mean ± s.d., $n = 2$, all individual data points are shown).

C *RBM3* E3a regulation is conserved in humans. HEK293 cells were incubated at the indicated temperatures for 12 h (DMSO/CHX last 4 h) and investigated for E3a inclusion as in B (mean ± s.d., $n = 3$, all individual data points are shown). For a representative gel image, see also Fig EV1A.

D Gene expression of *RBM3* anti-correlates with inclusion of E3a. Transcripts per million (TpM) values for *RBM3* are derived from RNA sequencing data from HEK293 cells incubated for the indicated time points for 12 h and are plotted on the right y-axis (green, $n = 2$, mean ± s.d.). Inclusion levels for E3a are derived from C.

E *RBM3* E3a stabilization in response to *SMG6* and *SMG7* knockdown and rescue (mean ± s.d., $n = 3$, for scrambled $n = 6$, all individual data points are shown) (Colombo *et al*, 2017). In all panels, statistical significance was determined by unpaired, two-sided t-test and is indicated by asterisks: P-values: $^*P < 0.05$, $^{**}P < 0.01$, $^{***}P < 0.001$, $^{****}P < 0.0001$.

Source data are available online for this figure.

globin sequences revealed two strong enhancer elements, both in HEK293 at 37 or 39°C (M2 and M4; Figs 3C and EV3B; Table EV1). We also identified silencer elements in the regions downstream of the 3'ss (M1), close to an internal 3'ss (M3) and upstream of the 5'ss (M6). In contrast to mutations of the M2 and M4 enhancer elements or deleting their evolutionary conserved core sequence, silencer mutants remained mostly temperature-sensitive (compare 37 and 39°C in Fig EV3B and C). This suggests that temperature sensitivity is mainly mediated by the enhancers, whereas the silencer elements rather control basal E3a inclusion levels, for example by controlling splice site accessibility. Deleting (instead of replacing) the M2 or M4 enhancer sequences also abolished E3a inclusion, and smaller replacements revealed the location of the enhancer elements within nucleotides 42–71 and 142–171 of the exon (Fig EV3D; Table EV1).

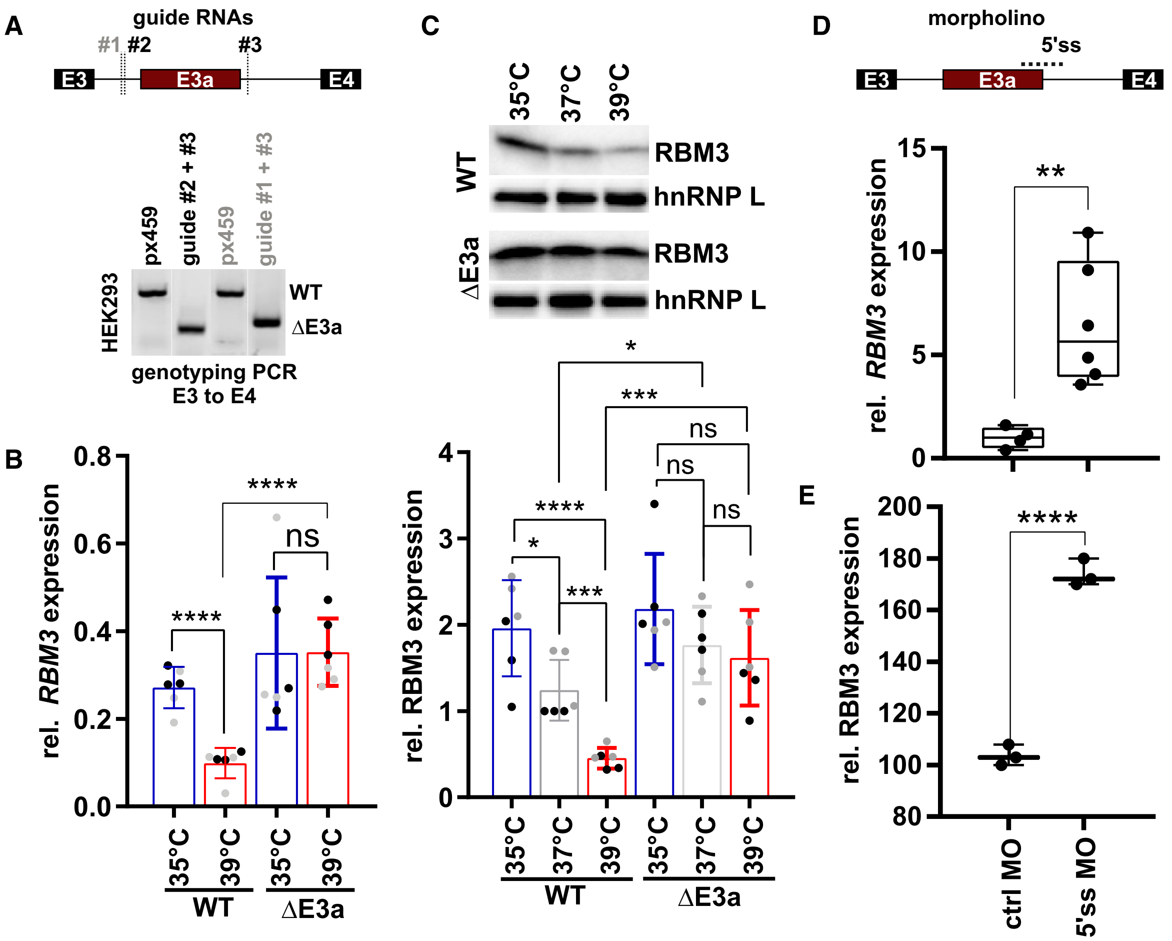

Figure 2. E3a controls temperature-dependent RBM3 expression.

A CRISPR/CAS9-mediated removal of *RBM3* E3a. One of two guide RNAs targeting the upstream intron (#1, #2) was co-transfected with a guide RNA targeting the downstream intron (#3). Below, genotyping PCR after clonal selection with px459 transfected cells serving as a negative control. See also Fig EV2A.

B, C RT–qPCR (B) and Western blot (C) analysis of *RBM3* levels in edited cell lines. Clonal cell lines from A were incubated at the indicated temperatures for 24 h. In (B), isolated RNA was investigated by qPCR, and *RBM3* expression is shown relative to *GAPDH* levels. In (C), lysates from an independent experiment were investigated for RBM3 protein expression, hnRNP L served as a loading control, a representative gel (top) and quantification (bottom) are shown (mean ± s.d., $n = 6$ [$n = 3$ per clone, indicated in black/gray], all individual data points are shown). See Fig EV2B for all gels.

D, E Manipulation of *RBM3* E3a splicing directly controls *RBM3* expression levels. In (D), HEK293 cells were transfected for 48 h at 37°C with a MO blocking the 5'ss of E3a. *RBM3* expression is shown relative to *GAPDH* levels and normalized to a non-targeting MO ($n = 4$–6, line indicates median, whiskers min to max, all individual data points are shown). In (E), primary hippocampal neurons were transfected with the indicated MOs for 48 h and investigated for RBM3 by Western blotting. GAPDH served as a loading control ($n = 3$; line indicates median, whiskers min to max, all individual data points are shown). Gel images in Fig EV2D. In all panels, statistical significance was determined by unpaired, two-sided *t*-test and is indicated by asterisks: *P*-values: $^*P < 0.05$, $^{**}P < 0.01$, $^{***}P < 0.001$, $^{****}P < 0.0001$.

Source data are available online for this figure.

As a basis for therapeutic manipulation of RBM3 levels in humans, we used this minigene analysis and performed an ASO screen targeting different cis-regulatory elements. We identified several ASOs targeting the M2 or M4 enhancers or the 5'ss that prevent E3a inclusion in HeLa cells (Figs 3D and EV4A–C; Table EV2). We noticed that ASOs targeting the M4 region or the 5'ss result in partial usage of internal alternative 3′ or 5′ splice sites leading to products that could still induce NMD. However, all variants targeting the evolutionary conserved M2D region (Fig EV4A) quantitatively abolish E3a inclusion (Fig EV4C), making them promising candidates for therapeutic applications. Importantly, the M2D ASO induces RBM3 expression at 40°C to the level observed in control cells at 37°C and is also inducing RBM3 expression up to twofold at 37°C (Fig 3E and F). M2D and M2Db also worked in a mouse cell line, as they induced *Rbm3* expression 1.5- (37°C) to 4 (39°C)-fold in N2a cells (Fig EV4D). In these experiments, we combined a phosphorothioate (PS)-modified backbone with uniform 2′-O-methoxyethyl (MOE)-modified bases as in the FDA-approved drug Nusinersen (Hua *et al*, 2011), which allows distribution throughout the central nervous system after intrathecal injection (Finkel *et al*, 2016) and systemic delivery *in vivo* (Sheng *et al*, 2020). To provide *in vivo* evidence that AS-NMD modulating ASOs can increase RBM3 levels in a therapeutic range in the central nervous system, we chose two ASOs targeting the M2 region, M2D and M2Db. We first checked for efficacy in RBM3 induction of the individual ASOs when administered by a single intracerebroventricular injection to wild-type mice

 © 2023 The Authors

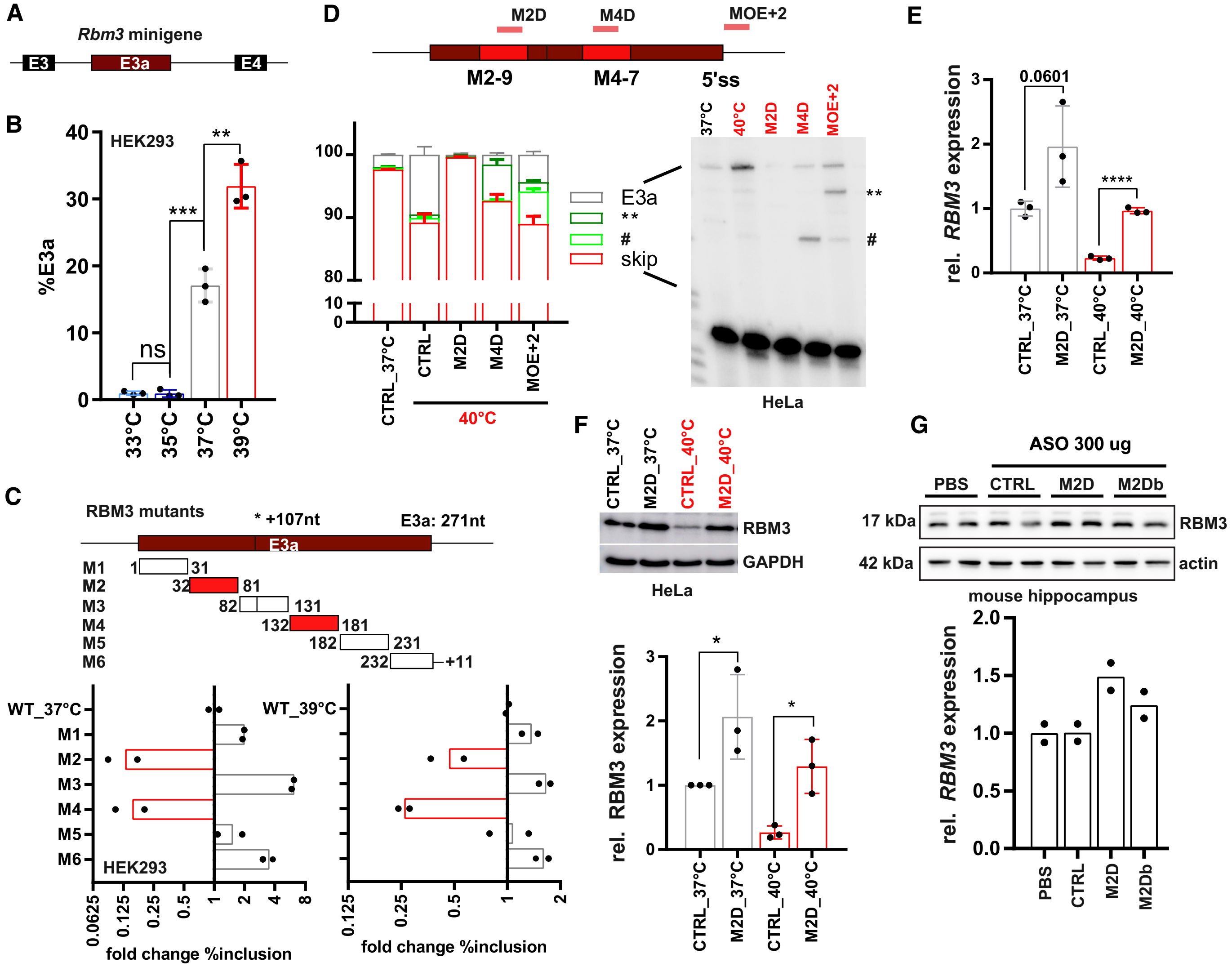

Figure 3. Targeting enhancer elements to control *RBM3* E3a inclusion.

A, B An *Rbm3* minigene reproduces temperature-controlled E3a inclusion. In (A), the minigene structure containing the whole unshorten sequence from E3 to E4 (including upstream 3′ss and downstream 5′ss) is shown. In (B), minigenes from mouse were transfected into HEK293 and incubated at the indicated temperatures for 12 h. E3a inclusion was investigated by splicing-sensitive PCR and quantified, %E3a is shown (mean ± s.d., $n = 3$, all individual data points are shown).

C Systematic mutational screening for regulatory elements. Mutations resulting in exon skipping are highlighted in red. Below, analysis of fold change in %E3a in HEK293 at 37 and 39°C (mean, $n = 2$, all individual data points are shown). Position 107 marks an alternative 3′ss in E3a. See also Fig EV3B and Table EV1.

D ASOs targeting M2-9, M4-7, or the 5′ss (see Fig EV4A and Table EV2) prevent endogenous *RBM3* E3a inclusion in human HeLa cells. ASO-transfected cells were kept for 24 h at 40°C. Control samples at 37 and 40°C are shown; CHX was added for the last 4 h. Exon 3a inclusion was investigated by splicing-sensitive RT–PCR, a representative gel and phosphorimager quantification are shown (mean ± s.d., $n = 3$). The hashtag marks the use of internal 5′ and 3′ss that is promoted by all ASOs targeting the M4 region. ASOs targeting the 5′ss induced the usage of an internal 5′ss (marked by two asterisks).

E, F M2D induces RBM3 mRNA (E) and protein (F) expression in human HeLa cells. ASOs were transfected for 24 h at 37°C (gray) or at 40°C (red). RBM3 induction was measured relative to GAPDH expression (mean ± s.d., $n = 3$, all individual data points are shown).

G M2D induces RBM3 protein expression *in vivo*. Hippocampus samples from two mice per condition were analyzed by Western blotting (left), and RBM3 protein was quantified relative to actin and PBS (right, mean ± s.d., $n = 2$, all individual data points are shown). In all panels, statistical significance was determined by unpaired, two-sided *t*-test and is indicated by asterisks: *P*-values: *$P < 0.05$, **$P < 0.01$, ***$P < 0.001$, ****$P < 0.0001$.

Source data are available online for this figure.

at doses of 100 and 300 μg. Both ASOs were well tolerated up to 3 weeks post-injection, and both increased RBM3 protein levels. M2D was the more efficient, resulting in 1.5-fold increase of RBM3 in the hippocampus at both 100 and 300 μg doses (Figs 3G and EV4E) and correlating with reduced E3a inclusion levels (Fig EV4F). We therefore focused on M2D for further testing *in vivo*.

We tested the therapeutic potential of M2D-mediated RBM3 induction in a mouse prion disease model extensively used to test the effects of cooling and RBM3 over-expression on the progression of neurodegeneration (Peretti *et al*, 2015, 2021; Bastide *et al*, 2017). Hemizygous tg37$^{+/-}$ mice overexpress prion protein (PrP) at around threefold over wild-type levels (Mallucci *et al*, 2002). When

© 2023 The Authors

inoculated with Rocky Mountain Laboratory (RML) prions, these mice show a rapid incubation time, succumbing to disease in only 12 weeks post-prion inoculation (w.p.i.), with rapidly progressing spongiform change and extensive neurodegeneration throughout the brain, including hippocampal CA1-3 regions (Mallucci *et al*, 2002). Early cooling (at 3 w.p.i.) to boost RBM3 levels, or lentiviral delivery of RBM3 to the hippocampus, are both profoundly neuroprotective in prion-diseased tg37$^{+/-}$ mice and in Alzheimer's 5xFAD mice, whereas RNAi of RBM3 eliminates the protective effects of cooling in both models (Peretti *et al*, 2015). We treated prion-diseased tg37$^{+/-}$ mice (n = 8) with 200 µg of the M2D ASO or a non-targeting control ASO (Fig 4A). This dose equates to ~ 8 µg/g in the

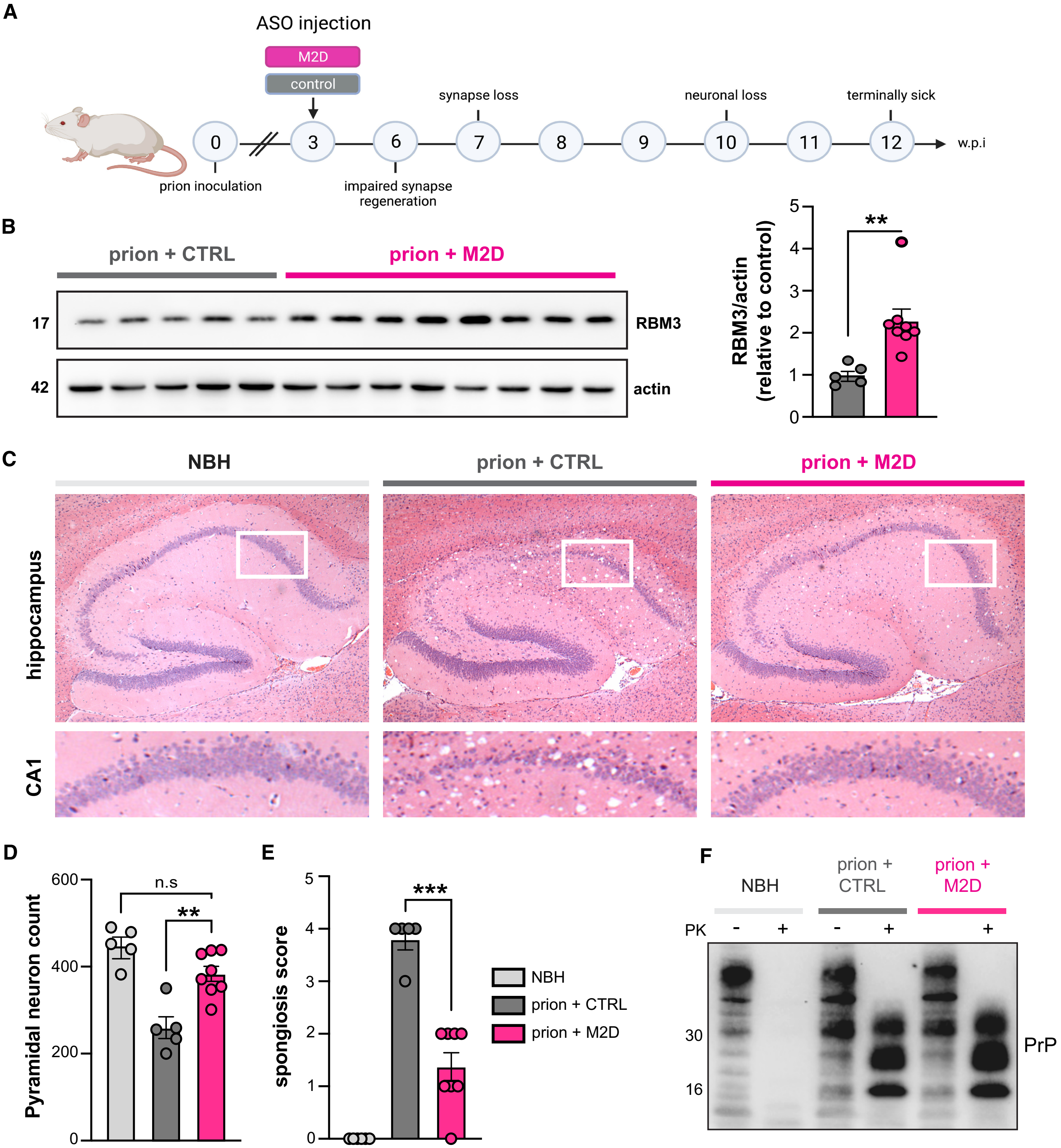

Figure 4.

 © 2023 The Authors

Figure 4. M2D elevates hippocampal RBM3 levels and is profoundly neuroprotective.

A Schematic of experimental design. Prion-inoculated tg37$^{+/-}$ mice were injected with 200 μg of either M2D or a non-targeting control ASO at 3 w.p.i.

B Western blot of hippocampal lysates from prion-infected mice treated with non-targeting control ASO (n = 5 mice) or M2D (n = 8 mice). M2D increases RBM3 expression by twofold compared to control ASO-treated mice, 9 weeks after ASO injection at 12 w.p.i. **P = 0.0058, calculated using Student's t-test.

C Representative images of hematoxylin and eosin-stained brain slices from NBH (control), and prion-infected control ASO- and M2D-treated mice at 12 w.p.i. when control ASO-treated mice were culled for prion signs. M2D confers marked neuroprotection in the hippocampus, with conservation of CA1-3 pyramidal layer, protection from shrinkage of the whole hippocampus, as well as reduced spongiform change.

D NeuN counts of pyramidal neurons in NBH (n = 5 mice) versus control ASO-treated (n = 5 mice) and M2D-treated prion mice (n = 8 mice). M2D confers neuroprotection close to levels seen in NBH mice. control ASO-treated versus M2D-treated prion mice **P = 0.0014. One-way ANOVA.

E Semi-quantitative scoring of spongiosis in NBH, control ASO-treated and M2D-treated mice. Sections showing no signs of spongiosis were scored 0, severe spongiosis was scored 4, as described (White *et al*, 2008). M2D (n = 8 mice) significantly reduced spongiosis compared to control ASO-treated prion mice (n = 5 mice), which is absent in uninfected NBH mice (n = 5 mice) ***P = 0.0001, one-way ANOVA.

F Total PrP and proteinase K-resistant PrP^{Sc} levels in NBH-injected mice and in prion-diseased mice injected with control—or M2D ASOs. PrP^{Sc} levels are unaffected by M2D-mediated RBM3 induction.

Source data are available online for this figure.

mouse which is similar to that used in SMA studies (Passini *et al*, 2011). ASOs were delivered by a single intracerebroventricular injection at 3 w.p.i., consistent with the timing of our previous interventions (Peretti *et al*, 2015, 2021; Bastide *et al*, 2017). Mice treated with M2D/non-targeting control ASO were analyzed for neuroprotection at 12 w.p.i. when all control-ASO-treated mice had succumbed to prion disease. No mice showed signs of toxicity associated with ASO administration throughout the experiment. Remarkably, the single dose of M2D ASO resulted in RBM3 levels twofold higher than control ASO-treated mice 9 weeks after injection, at 12 w.p.i. (Fig 4B). Higher levels of RBM3 were associated with marked neuroprotection: 7/8 M2D-treated mice showed extensive preservation of pyramidal neurons in the hippocampal CA1-3 regions (Fig 4C and D) compared to control-ASO-treated mice, of which 5 of 6 mice showed profound neuronal loss in these regions (Fig 4C and D). M2D-mediated neuroprotection was associated with markedly lower spongiosis scores compared to control ASO-treated mice (Fig 4E) and did not alter total levels of PrP or of the amyloid form, proteinase K-resistant PrP^{Sc} (Fig 4F). These data support a robust and long-lasting induction of RBM3 without cooling by a single dose of M2D, with remarkable neuroprotection in the context of a rapidly progressive neurodegenerative disorder. The ability to raise RBM3 levels by one administration of a well-tolerated ASO using FDA-approved chemistry, in place of therapeutic hypothermia, has strong implications for neuroprotection in diverse conditions, from acute treatment of neonates through to cardiac surgery, stroke, and head injury in adults. In the acute setting, this approach yields the neuroprotective effect of RBM3 while bypassing the substantial risks associated with intensive care and hypothermia. Further, the approach has marked appeal in the potential prevention of a variety of neurodegenerative disorders. ASOs are highly successful in children with SMA and have been recently licensed for the treatment of the rapidly progressive adult neurodegenerative disorder, ALS (Miller *et al*, 2022). While the approach to SMA therapy, which—like ours—involves ASO-mediated splicing modulation, has proved highly successful in human disease, ASO use has been problematic in some areas. Notably, ASOs in Huntington's disease—albeit being directed at reducing total mRNA expression levels—have not been successful in early human trials, for reasons that are not clear (Kingwell, 2021). Safety and large animal studies will be needed before RBM3-inducing ASOs could be trialed in humans, of course, as well as continually exploring alternative means of RBM3 induction (Ávila-Gómez *et al*, 2022) and reviewing any possible role of RBM3 in tumor growth and protection (Zhou *et al*, 2017). However, in the search for disease-modifying therapies for Alzheimer's disease and related dementias, induction of the broadly neuroprotective protein RBM3 via ASO delivery to drive its long-term expression is a compelling therapeutic approach. Increasing RBM3 expression boosts neuronal resilience and synapse regeneration that are key to resisting the direct and indirect toxic effects of protein misfolding disorders, particularly in the context of age-related dementias.

Materials and Methods

RNA-Seq analysis and bioinformatics

RNA sequencing data from mouse primary hepatocytes were obtained from GSE158882 (Neumann *et al*, 2020). Sequencing data from HeLa cells after knockdown and rescue of *UPF1*, *SMG6*, and *SMG7* were obtained from SRP083135 (Colombo *et al*, 2017). Mapping of reads to reference genomes (mm10 for mouse, hg38 for human) was performed using STAR version 2.5.3a (Dobin *et al*, 2012). The RBM3 Sashimi plot was generated using a customized version of ggsashimi (Garrido-Martin *et al*, 2018), which additionally displays conservation scores. Percent spliced in values for *Rbm3* E3a after knockdown and depletion was manually calculated from junction read counts.

Tissue culture cells

HEK293T, HeLa (both human), and N2a (mouse) cell stocks are maintained in liquid nitrogen, and early passage aliquots are thawed periodically. To rule out mycoplasma contamination, cell morphology is routinely assessed and monthly checked using a PCR-based assay. HEK293T and HeLa cells were cultured in DMEM high glucose with 10% FBS and 1% penicillin/ streptomycin. N2a cells were cultured in 50% Opti-MEM/50% Opti-MEM GlutaMAX with 10% FBS and 1% penicillin/streptomycin. Mouse hippocampal neurons were isolated and maintained as previously described (Peretti *et al*, 2021). All cell lines were usually maintained at 37°C and 5% CO_2. For temperature experiments, cells were shifted into pre-equilibrated incubators for the indicated times. For square-wave temperature cycles, we used two incubators set to 34 and 38°C and shifted the cells every 12 h (Preussner *et al*, 2017). Transfections of HEK293T or N2a cells (for minigenes or CRISPR guides) using ROTI-Fect (Roth) were performed according to the manufacturer's

© 2023 The Authors

instructions. Cycloheximide (Sigma) was used at 40 μg/ml final concentration or DMSO as solvent control. For morpholino experiments, cells were seeded and transfected 1 day later using Endo-Porter following the manufacturer's manual. Morpholinos (*RBM3* 5'ss: GTCTCCCCTGCTACTACTTACATCT and standard control) and Endo-Porter transfection reagent were purchased from Gene Tools. MOE antisense oligonucleotides (1 μl, 100 ng/μl) were purchased from Microsynth (see Table EV2 for sequences) and transfected into Hela or N2a cells ($3*10^6$ cells per well on a 12-well plate) via ROTI-Fect, and 4 h later, the transfected cells were transferred to incubators with the indicated temperature, followed by RNA extraction 24 h later and RT–qPCR or splicing-sensitive PCR.

RT–PCR and RT–qPCR

RT–PCRs were done as previously described (Preussner *et al*, 2014). Shortly, RNA was extracted using RNATri (Bio&Sell) and 1 μg RNA was used in a gene-specific RT reaction. Endogenous *RBM3* splicing was analyzed with a radioactively labeled forward primer in exon 3 (5′-TCATCACCTTCACCAACCCA) and a reverse primer in exon 5 (5′-TCTAGAGTAGCTGCGACCAC). For analysis of minigene splicing, the RNA was additionally digested with DNase I and re-purified. Minigene splicing was investigated with minigene-specific primers: T7fwd: 5′-GACTCACTATAGGGAGACCC; BGHrev: 5′-TAGAAGGCACAGTCGAGG. In some cases, a minigene-specific RT with BGHrev was followed by a PCR with forward primer targeting exon 3 (5′-TGGTTGTTGTCAAGGACCGGG) and reverse primer targeting exon 5 (5′-CTCTAGAGTAACTGCGACCAC). For RT–qPCR, the *RBM3* gene-specific primer was combined with a housekeeping gene reverse primer in one RT reaction. qPCR was then performed in a 96-well format using the Blue S'Green qPCR Kit Separate ROX (Biozym) on Stratagene Mx3000P instruments. qPCRs were performed in technical duplicates, mean values were used to normalize expression to a housekeeping gene (human: GAPDH; mouse: HPRT); DCT and D(DCT)s were calculated for different conditions.

mRBM3_E2/3_qF	AAGGGAAACTCTTCGTAG	qPCR mouse
mRBM3_E3_qR	GACAACAACCACCTCAGAGATAG	qPCR mouse
HPRT_qF	CAACGGGGGACATAAAAGTTATTGGTGGA	qPCR mouse
HPRT_qR	TGCAACCTTAACCATTTTGGGGCTGT	qPCR mouse
hRBM3_E2/3_qF	AAGGAAAGCTCTTCGTGGGA	qPCR human
hRBM3_E3_qR	GACAACGACCACCTCAGAGA	qPCR human
GAPDH_qF	CTTCGCTCTCTGCTCCTCCTGTTCG	qPCR human
GAPDH_qR	ACCAGGCGCCCAATACGACCAAAT	qPCR human

Generation of CRISPR-Cas9 edited cells and analysis of RBM3 expression

For genome-engineering in HEK293 cells, sequences flanking E3a of *RBM3* were analyzed for sgRNA candidates *in silico* using the Benchling tool. A pair of oligonucleotides for the highest ranked candidate sgRNA (Ran *et al*, 2013) upstream and downstream of the exon was synthesized and subcloned into the PX459 vector. sgRNA sequences #1: 5′-TGTGTCTGCTCGGGGCAGCG; #2: 5′-CCTGTGAG TGGGCACTGCG; #3: 5′-TCCTGATGAAGCCATTCTG. Cells were co-transfected with guide RNA #3 and either #1 or #2 in 6-well plates using ROTIFect (Roth) according to the manufacturer's instructions. 48 h after transfection, the transfected cells were selected with 1 μg/ml puromycin and clonal cell lines were isolated by dilution. Genomic DNA was extracted using DNA extraction buffer (200 mM Tris–HCl pH 8.3, 500 mM KCl, 5 mM $MgCl_2$, 0.1% gelatin in H_2O) and to confirm the exon knockout on DNA level, a genotyping PCR was performed using primers binding in introns upstream and downstream of the cutting sites (FWD: 5′-ATCTGCAGAGGGACCTTGTC; REV: 5′-CAGACTTGCCTGCATGATCC). In promising clones, the exon knockout was additionally confirmed after RNA isolation by splicing-sensitive PCR using one forward primer in exon 3 and one reverse primer in exon 4. RBM3 total expression levels were investigated by RT–qPCR and Western blot.

Western blot

Whole-cell extracts (WCEs) were prepared with lysis buffer (20 mM Tris [pH 8.0], 2% NP-40 [v/v], 0.01% sodium deoxycholate [w/v], 4 mM EDTA and 200 mM NaCl) supplemented with protease inhibitor mix (Aprotinin, Leupeptin, Vanadate, and PMSF). Concentrations were determined using Roti Nanoquant (Roth), according to the manufacturer's instructions. Mouse tissue was lysed in RIPA buffer (5-mM Tris [pH 8.0], 150 mM sodium chloride, 1% IGEPEL, 0.5% sodium deoxycholate, 0.1% sodium dodecyl sulfate) supplemented with protease and phosphatase inhibitors (Roche). SDS–PAGE and Western blotting followed standard procedures. Western blots were quantified using the ImageQuant TL software. The following antibodies were used for Western blotting: hnRNPL (4D11, Santa Cruz), RBM3 (Proteintech), GAPDH (GT239, GeneTex), actin (4970, Cell Signaling), PrP (ICSM35, D-Gen).

Minigene constructs

Cloning was performed using PCR introducing HindIII and XhoI sites and ligation into pcDNA3.1 (+). Constructs were cloned using a forward primer in the intron upstream of exon 3 (mouse: 5′-AACTTAAGCTTCTGTGGCTGTGCCTGGCT; human: 5′-AACTT AAGCTTTCCGGCCAC CCTTTGCTAC), a reverse primer in the intron downstream of exon 4 (mouse: 5′-CTAGACTCCAGTT CAGACATAGGCTCTTAA; human: 5′-TAGACTCGAGATAGGCAACT CTCCC TCTCA) and human or mouse DNA as template. For each of mRRM3 mutant (see Table EV1) and sub-mutant cloning, the mutated sequences were deleted or replaced by sequences from human beta-globin (M3 contains a 3'ss, M6 a 5'ss, and the remaining mutants exon 2 sequences). Briefly, two DNA fragments were amplified from the mRBM3 minigene with PCR primer pairs introducing the mutation. Then, these two DNA fragments were used as templates for a PCR to get the full-length mRBM3 mutants. All minigene sequences were confirmed by sequencing (Microsynth Seqlab).

© 2023 The Authors

Primary neuronal culture

Primary neurons were isolated from the hippocampi of both male and female C57Bl6/N mouse pups at post-natal day 0 or 1. For the isolation and culture of hippocampal neurons, we followed the protocol of Beaudoin *et al* (2012) with slight modifications. Briefly, hippocampi were extracted into Hibernate A media (Gibco) and incubated at 37°C with papain solution for 20 min. Papain solution was removed, and trypsin inhibitor was added for 5 min. Hippocampi were then washed 3 times in pre-warmed plating media (Neurobasal A, B27 supplement, GlutaMAX, Horse serum, 1 M HEPES pH 7.5) before being triturated 8–10 times. The suspension was strained, and 800,000 cells were seeded onto 6-well plates coated with poly-L-lysine. Media was changed to neuron media (Neurobasal A, B27 supplement, GlutaMAX, Penicillin/Streptomycin) 4 h post-seeding. Primary neurons were maintained at 37°C, 5% CO_2 or as indicated at 32°C. 5-fluoro-2′-deoxyuridine (Sigma-Aldrich) was added at a final concentration of 7.15 µg/ml to inhibit glial growth (Vossel *et al*, 2015). A third of the media was changed for fresh media every 4–5 days. Experimental procedures lasting 24–48 h were started at day 19–20 D.I.V. to finish at 21 D.I.V.

Mice

All animal work conformed to UK Home Office regulations and were performed under the Animal [Scientific Procedures] Act 1986, Amendment Regulations 2012 and following institutional guidelines for the care and use of animals for research. All studies were ethically reviewed by the University of Cambridge Animal Welfare and Ethical Review Body (AWERB). Mice were housed in groups of 2–5 animals/cage, under a 12 h light/dark cycle, and were tested in the light phase. Water and standard mouse chow were given ad-libitum. Mice were randomly assigned treatment groups by cage number. Experimenters were blind to group allocation during the experiments and when assessing clinical signs. Procedures were fully compliant with Animal Research: Reporting of *In Vivo* Experiments (ARRIVE) guidelines.

Prion infection of mice

Male and female 3-week-old tg37$^{+/-}$ mice (Tg(Prnp)37Jcol) (Mallucci *et al*, 2002) were inoculated intra-cerebrally into the right parietal lobe with 30 µl 1% brain homogenate of Chandler/RML (Rocky Mountain Laboratories) prions under general anesthetic, as described (Mallucci *et al*, 2002). Animals were culled when they developed clinical signs of scrapie as defined in (Mallucci *et al*, 2002, 2003, 2007). Control mice received 1% normal brain homogenate.

Administration of ASOs

6-week-old uninoculated tg37 hemizygous mice were injected with 100 or 300 µg of non-targeting control ASO or M2D ASO. ASOs were administered via intracerebroventricular injection at −0.3Y, 1.0X, 3.0Z anterior to bregma. Mice were culled 3 weeks post-injection. Prion-infected mice were injected with 200 µg of non-targeting control ASO or M2D ASO at 3 w.p.i and culled at 12 w.p.i. This dose was chosen as an average of the efficacious doses of 100 and 300 µg. All surgeries were performed under general anesthesia. Both males and females were used, with random allocation to experimental groups.

The paper explained

Problem

Neurodegenerative diseases, including Alzheimer's and related dementias, are now the leading cause of death in the developed world—and are rapidly increasing in the developing world. To date, there are no treatments or interventions that meaningfully slow or prevent disease. As the global population ages, these diseases will place an ever-increasing, overwhelming burden on families, health care systems, and economies worldwide. New treatments are urgently required to modify disease progression. Pre-clinical data have shown that inducing the cold-shock protein, RBM3 by hypothermia regenerates synapses, prevents loss of brain cells, boosts memory, and significantly prolonging disease-free survival in mouse models of neurodegeneration.

Results

In this study, Preussner, Smith and colleagues have discovered a means of inducing RBM3 without systemic cooling. They identified a "poison" exon within RBM3 pre-mRNA, the exclusion of which enables RBM3 protein expression during cooling. Using FDA-approved chemistry, they generated an ASO targeting a splice-regulatory sequence of the poison exon, and this ASO increases RBM3 expression at normothermia. A single intrathecal dose of the ASO in mice with the rapid neurodegenerative disorder, prion disease, was sufficient to stably induce RBM3 expression over 9 weeks and prevent spongiform change and neuronal loss in diseased mice.

Impact

ASO treatment targeting splicing in human neurodegenerative disorders such as spinal muscular atrophy is highly successful, and trials of ASOs are underway for ALS and other disorders. ASO-mediated RBM3 induction, in contrast, is a disease-agnostic approach that could prove neuroprotective across the spectrum of neurodegenerative disorders. It could also be used—instead of cooling—in the acute scenario of therapeutic hypothermia-treated disorders ranging from birth asphyxia to stroke to coronary care. The clinical impact, if effective in humans, would be transformative in both neurodegenerative disease and acute brain injury.

Histology

Paraffin-embedded brains were sectioned at 5 mm and stained with H&E as described (Moreno *et al*, 2012). Neuronal counts were determined by quantifying NeuN-positive pyramidal CA1 neurons as described (Moreno *et al*, 2012).

Statistical analysis

Data are presented as the mean ± standard error of the mean (SEM) of biological replicates unless otherwise specified in the legend. Statistical significance was determined using GraphPad Prism v7 or v8, using a Student's *t*-test or one-way ANOVA with Tukey's test for multiple comparisons for normally distributed data or Kruskal–Wallis test and Dunn's multiple comparisons test for non-normally distributed data sets. Statistical significance was accepted at $P \le 0.05$. In the figure legends, "ns" denotes $P \ge 0.05$, * denotes $P \le 0.05$, ** denotes $P \le 0.01$, ***$P \le 0.001$ and **** denotes $P \le 0.0001$.

Data availability

This study includes no data deposited in external repositories.

Expanded View for this article is available online.

Acknowledgements

The authors would like to thank HPC Service of ZEDAT, Freie Universität Berlin, for computing time. We thank members of the Heyd and Mallucci labs for constructive discussions and comments. This work was supported by core funding from the Freie Universität Berlin (to F.H.) and by UK Dementia Research Institute (award UKDRI-2001), which receives its funding from UK DRI Ltd (funded by the UK Medical Research Council, Alzheimer's Society and Alzheimer's Research UK) and by Medical Research Council (award MRC MC_U132692719) (to G.R.M.). Additional funding was provided by the Deutsche Forschungsgemeinschaft (grants HE5398/4-2 to F.H.) and the Cambridge Centre for Parkinson's Plus (D.H.). Open Access funding enabled and organized by Projekt DEAL.

Author contributions

Marco Preußner: Conceptualization; data curation; formal analysis; supervision; validation; investigation; visualization; methodology; writing – original draft; writing – review and editing. **Heather L Smith:** Data curation; formal analysis; validation; investigation; visualization; methodology; writing – original draft; writing – review and editing. **Daniel Hughes:** Investigation. **Min Zhang:** Investigation. **Ann-Kathrin Emmerichs:** Investigation. **Silvia Scalzitti:** Investigation. **Diego Peretti:** Investigation. **Dean Swinden:** Investigation. **Alexander Neumann:** Investigation. **Tom Halteenhof:** Investigation. **Giovanna R Mallucci:** Conceptualization; resources; data curation; formal analysis; supervision; funding acquisition; visualization; methodology; writing – original draft; project administration; writing – review and editing. **Florian Heyd:** Conceptualization; resources; data curation; formal analysis; supervision; funding acquisition; visualization; methodology; writing – original draft; project administration; writing – review and editing.

Disclosure and competing interests statement

A patent application has been filed in relation to this research. There are no other competing interests.

References

Ávila-Gómez P, Pérez-Mato M, Hervella P, Dopico-López A, Silva-Candal AD, Bugallo-Casal A, López-Amoedo S, Candamo-Lourido M, Sobrino T, Iglesias-Rey R *et al* (2022) Associations between RNA-binding motif protein 3, fibroblast growth factor 21, and clinical outcome in patients with stroke. *J Clin Med* 11: 949

Azzopardi D, Strohm B, Marlow N, Brocklehurst P, Deierl A, Eddama O, Goodwin J, Halliday HL, Juszczak E, Kapellou O *et al* (2014) Effects of hypothermia for perinatal asphyxia on childhood outcomes. *N Engl J Med* 371: 140–149

Bastide A, Peretti D, Knight JR, Grosso S, Spriggs RV, Pichon X, Sbarrato T, Roobol A, Roobol J, Vito D *et al* (2017) RTN3 is a novel cold-induced protein and mediates neuroprotective effects of RBM3. *Curr Biol* 27: 638–650

Beaudoin GM 3rd, Lee SH, Singh D, Yuan Y, Ng YG, Reichardt LF, Arikkath J (2012) Culturing pyramidal neurons from the early postnatal mouse hippocampus and cortex. *Nat Protoc* 7: 1741–1754

Bennett CF, Krainer AR, Cleveland DW (2019) Antisense oligonucleotide therapies for neurodegenerative diseases. *Annu Rev Neurosci* 42: 385–406

Bernard SA, Smith K, Finn J, Hein C, Grantham H, Bray JE, Deasy C, Stephenson M, Williams TA, Straney LD *et al* (2016) Induction of therapeutic hypothermia during out-of-hospital cardiac arrest using a rapid infusion of cold saline: the RINSE trial (rapid infusion of cold normal saline). *Circulation* 134: 797–805

Chip S, Zelmer A, Ogunshola OO, Felderhoff-Mueser U, Nitsch C, Bührer C, Wellmann S (2011) The RNA-binding protein RBM3 is involved in hypothermia induced neuroprotection. *Neurobiol Dis* 43: 388–396

Ciuzan O, Hancock J, Pamfil D, Wilson I, Ladomery M (2015) The evolutionarily conserved multifunctional glycine-rich RNA-binding proteins play key roles in development and stress adaptation. *Physiol Plant* 153: 1–11

Colombo M, Karousis ED, Bourquin J, Bruggmann R, Mühlemann O (2017) Transcriptome-wide identification of NMD-targeted human mRNAs reveals extensive redundancy between SMG6- and SMG7-mediated degradation pathways. *RNA* 23: 189–201 [DATASET]

Dankiewicz J, Cronberg T, Lilja G, Jakobsen JC, Levin H, Ullén S, Rylander C, Wise MP, Oddo M, Cariou A *et al* (2021) Hypothermia versus normothermia after out-of-hospital cardiac arrest. *N Engl J Med* 384: 2283–2294

Danno S, Nishiyama H, Higashitsuji H, Yokoi H, Xue JH, Itoh K, Matsuda T, Fujita J (1997) Increased transcript level of RBM3, a member of the glycine-rich RNA-binding protein family, in human cells in response to cold stress. *Biochem Biophys Res Commun* 236: 804–807

Dobin A, Davis CA, Schlesinger F, Drenkow J, Zaleski C, Jha S, Batut P, Chaisson M, Gingeras TR (2012) STAR: ultrafast universal RNA-seq aligner. *Bioinformatics* 29: 15–21

Erdos MR, Cabral WA, Tavarez UL, Cao K, Gvozdenovic-Jeremic J, Narisu N, Zerfas PM, Crumley S, Boku Y, Hanson G *et al* (2021) A targeted antisense therapeutic approach for Hutchinson-Gilford progeria syndrome. *Nat Med* 27: 536–545

Finkel RS, Chiriboga CA, Vajsar J, Day JW, Montes J, De Vivo DC, Yamashita M, Rigo F, Hung G, Schneider E *et al* (2016) Treatment of infantile-onset spinal muscular atrophy with nusinersen: a phase 2, open-label, dose-escalation study. *Lancet* 388: 3017–3026

Garrido-Martin D, Palumbo E, Guigo R, Breschi A (2018) Ggsashimi: sashimi plot revised for browser- and annotation-independent splicing visualization. *PLoS Comput Biol* 14: e1006360

Haltenhof T, Kotte A, De Bortoli F, Schiefer S, Meinke S, Emmerichs AK, Petermann KK, Timmermann B, Imhof P, Franz A *et al* (2020) A conserved kinase-based body temperature sensor globally controls alternative splicing and gene expression. *Mol Cell* 78: 57–69.e4

Hoekstra MM, Emmenegger Y, Hubbard J, Franken P (2019) Cold-inducible RNA-binding protein (CIRBP) adjusts clock-gene expression and REM-sleep recovery following sleep deprivation. *Elife* 8: e43400

Hua Y, Sahashi K, Rigo F, Hung G, Horev G, Bennett CF, Krainer AR (2011) Peripheral SMN restoration is essential for long-term rescue of a severe spinal muscular atrophy mouse model. *Nature* 478: 123–126

Hypothermia after Cardiac Arrest Study Group (2002) Mild therapeutic hypothermia to improve the neurologic outcome after cardiac arrest. *N Engl J Med* 346: 549–556

Jackson TC, Manole MD, Kotermanski SE, Jackson EK, Clark RSB, Kochanek PM (2015) Cold stress protein RBM3 responds to temperature change in an ultra-sensitive manner in young neurons. *Neuroscience* 305: 268–278

Kingwell K (2021) Double setback for ASO trials in Huntington disease. *Nat Rev Drug Discov* 20: 412–413

© 2023 The Authors

Lascarrou JB, Merdji H, Le Gouge A, Colin G, Grillet G, Girardie P, Coupez E, Dequin PF, Cariou A, Boulain T *et al* (2019) Targeted temperature management for cardiac arrest with nonshockable rhythm. *N Engl J Med* 381: 2327 – 2337

Lim KH, Han Z, Jeon HY, Kach J, Jing E, Weyn-Vanhentenryck S, Downs M, Corrionero A, Oh R, Scharner J *et al* (2020) Antisense oligonucleotide modulation of non-productive alternative splicing upregulates gene expression. *Nat Commun* 11: 3501

Lin JQ, Khuperkar D, Pavlou S, Makarchuk S, Patikas N, Lee FCY, Kang J, Field SF, Zbiegly JM, Freeman JL *et al* (2022) HNRNPH1 regulates the neuroprotective cold-shock protein RBM3 expression through poison exon exclusion. *bioRxiv* https://doi.org/10.1101/2022.10.27.514062 [PREPRINT]

Liu Y, Hu W, Murakawa Y, Yin J, Wang G, Landthaler M, Yan J (2013) Cold-induced RNA-binding proteins regulate circadian gene expression by controlling alternative polyadenylation. *Sci Rep* 3: 2054

Los B, Preußner M, Eschke K, Vidal RM, Abdelgawad A, Olofsson D, Keiper S, Paulo-Pedro M, Grindel A, Meinke S *et al* (2022) Body temperature variation controls pre-mRNA processing and transcription of antiviral genes and SARS-CoV-2 replication. *Nucleic Acids Res* 50: 6769 – 6785

Lujan DA, Ochoa JL, Hartley RS (2018) Cold-inducible RNA binding protein in cancer and inflammation. *Wiley Interdiscip Rev RNA* 9:

Lykke-Andersen S, Jensen TH (2015) Nonsense-mediated mRNA decay: an intricate machinery that shapes transcriptomes. *Nat Rev Mol Cell Biol* 16: 665 – 677

Mallucci GR, Ratté S, Asante EA, Linehan J, Gowland I, Jefferys JG, Collinge J (2002) Post-natal knockout of prion protein alters hippocampal CA1 properties, but does not result in neurodegeneration. *EMBO J* 21: 202 – 210

Mallucci G, Dickinson A, Linehan J, Klöhn PC, Brandner S, Collinge J (2003) Depleting neuronal PrP in prion infection prevents disease and reverses spongiosis. *Science* 302: 871 – 874

Mallucci GR, White MD, Farmer M, Dickinson A, Khatun H, Powell AD, Brandner S, Jefferys JG, Collinge J (2007) Targeting cellular prion protein reverses early cognitive deficits and neurophysiological dysfunction in prion-infected mice. *Neuron* 53: 325 – 335

Miller TM, Cudkowicz ME, Genge A, Shaw PJ, Sobue G, Bucelli RC, Chiò A, Van Damme P, Ludolph AC, Glass JD *et al* (2022) Trial of antisense oligonucleotide Tofersen for SOD1 ALS. *N Engl J Med* 387: 1099 – 1110

Moreno JA, Radford H, Peretti D, Steinert JR, Verity N, Martin MG, Halliday M, Morgan J, Dinsdale D, Ortori CA *et al* (2012) Sustained translational repression by eIF2α-P mediates prion neurodegeneration. *Nature* 485: 507 – 511

Morf J, Rey G, Schneider K, Stratmann M, Fujita J, Naef F, Schibler U (2012) Cold-inducible RNA-binding protein modulates circadian gene expression posttranscriptionally. *Science* 338: 379 – 383

Neumann A, Meinke S, Goldammer G, Strauch M, Schubert D, Timmermann B, Heyd F, Preußner M (2020) Alternative splicing coupled mRNA decay shapes the temperature-dependent transcriptome. *EMBO Rep* 21: e51369

Nielsen N, Wetterslev J, Cronberg T, Erlinge D, Gasche Y, Hassager C, Horn J, Hovdenes J, Kjaergaard J, Kuiper M *et al* (2013) Targeted temperature management at 33°C versus 36°C after cardiac arrest. *N Engl J Med* 369: 2197 – 2206

Nishiyama H, Itoh K, Kaneko Y, Kishishita M, Yoshida O, Fujita J (1997) A glycine-rich RNA-binding protein mediating cold-inducible suppression of mammalian cell growth. *J Cell Biol* 137: 899 – 908

Passini MA, Bu J, Richards AM, Kinnecom C, Sardi SP, Stanek LM, Hua Y, Rigo F, Matson J, Hung G *et al* (2011) Antisense oligonucleotides delivered to the mouse CNS ameliorate symptoms of severe spinal muscular atrophy. *Sci Transl Med* 3: 72ra18

Peretti D, Bastide A, Radford H, Verity N, Molloy C, Martin MG, Moreno JA, Steinert JR, Smith T, Dinsdale D *et al* (2015) RBM3 mediates structural plasticity and protective effects of cooling in neurodegeneration. *Nature* 518: 236 – 239

Peretti D, Smith HL, Verity N, Humoud I, de Weerd L, Swinden DP, Hayes J, Mallucci GR (2021) TrkB signaling regulates the cold-shock protein RBM3-mediated neuroprotection. *Life Sci Alliance* 4: e202000884

Preussner M, Wilhelmi I, Schultz AS, Finkernagel F, Michel M, Moroy T, Heyd F (2014) Rhythmic U2af26 alternative splicing controls PERIOD1 stability and the circadian clock in mice. *Mol Cell* 54: 651 – 662

Preussner M, Goldammer G, Neumann A, Haltenhof T, Rautenstrauch P, Muller-McNicoll M, Heyd F (2017) Body temperature cycles control rhythmic alternative splicing in mammals. *Mol Cell* 67: 433 – 446.e4

Puttaraju M, Jackson M, Klein S, Shilo A, Bennett CF, Gordon L, Rigo F, Misteli T (2021) Systematic screening identifies therapeutic antisense oligonucleotides for Hutchinson-Gilford progeria syndrome. *Nat Med* 27: 526 – 535

Qiang X, Yang WL, Wu R, Zhou M, Jacob A, Dong W, Kuncewitch M, Ji Y, Yang H, Wang H *et al* (2013) Cold-inducible RNA-binding protein (CIRP) triggers inflammatory responses in hemorrhagic shock and sepsis. *Nat Med* 19: 1489 – 1495

Ran FA, Hsu PD, Wright J, Agarwala V, Scott DA, Zhang F (2013) Genome engineering using the CRISPR-Cas9 system. *Nat Protoc* 8: 2281 – 2308

Rosenthal LM, Leithner C, Tong G, Streitberger KJ, Krech J, Storm C, Schmitt KRL (2019) RBM3 and CIRP expressions in targeted temperature management treated cardiac arrest patients-a prospective single center study. *PLoS One* 14: e0226005

Shankaran S (2012) Hypoxic-ischemic encephalopathy and novel strategies for neuroprotection. *Clin Perinatol* 39: 919 – 929

Shankaran S, Laptook AR, Ehrenkranz RA, Tyson JE, McDonald SA, Donovan EF, Fanaroff AA, Poole WK, Wright LL, Higgins RD *et al* (2005) Whole-body hypothermia for neonates with hypoxic-ischemic encephalopathy. *N Engl J Med* 353: 1574 – 1584

Sheng L, Rigo F, Bennett CF, Krainer AR, Hua Y (2020) Comparison of the efficacy of MOE and PMO modifications of systemic antisense oligonucleotides in a severe SMA mouse model. *Nucleic Acids Res* 48: 2853 – 2865

Thayyil S, Pant S, Montaldo P, Shukla D, Oliveira V, Ivain P, Bassett P, Swamy R, Mendoza J, Moreno-Morales M *et al* (2021) Hypothermia for moderate or severe neonatal encephalopathy in low-income and middle-income countries (HELIX): a randomised controlled trial in India, Sri Lanka, and Bangladesh. *Lancet Glob Health* 9: e1273 – e1285

Vossel KA, Xu JC, Fomenko V, Miyamoto T, Suberbielle E, Knox JA, Ho K, Kim DH, Yu GQ, Mucke L (2015) Tau reduction prevents Aβ-induced axonal transport deficits by blocking activation of GSK3β. *J Cell Biol* 209: 419 – 433

White MD, Farmer M, Mirabile I, Brandner S, Collinge J, Mallucci GR (2008) Single treatment with RNAi against prion protein rescues early neuronal dysfunction and prolongs survival in mice with prion disease. *Proc Natl Acad Sci USA* 105: 10238 – 10243

Yang HJ, Zhuang RJ, Li YB, Li T, Yuan X, Lei BB, Xie YF, Wang M (2019) Cold-inducible protein RBM3 mediates hypothermic neuroprotection against neurotoxin rotenone via inhibition on MAPK signalling. *J Cell Mol Med* 23: 7010 – 7020

Zhou RB, Lu XL, Zhang CY, Yin DC (2017) RNA binding motif protein 3: a potential biomarker in cancer and therapeutic target in neuroprotection. *Oncotarget* 8: 22235 – 22250

© 2023 The Authors

Zhu X, Yan J, Bregere C, Zelmer A, Goerne T, Kapfhammer JP, Guzman R, Wellmann S (2019) RBM3 promotes neurogenesis in a niche-dependent manner via IMP2-IGF2 signaling pathway after hypoxic-ischemic brain injury. *Nat Commun* 10: 3983

License: This is an open access article under the terms of the Creative Commons Attribution License, which permits use, distribution and reproduction in any medium, provided the original work is properly cited.

© 2023 The Authors

Expanded View Figures

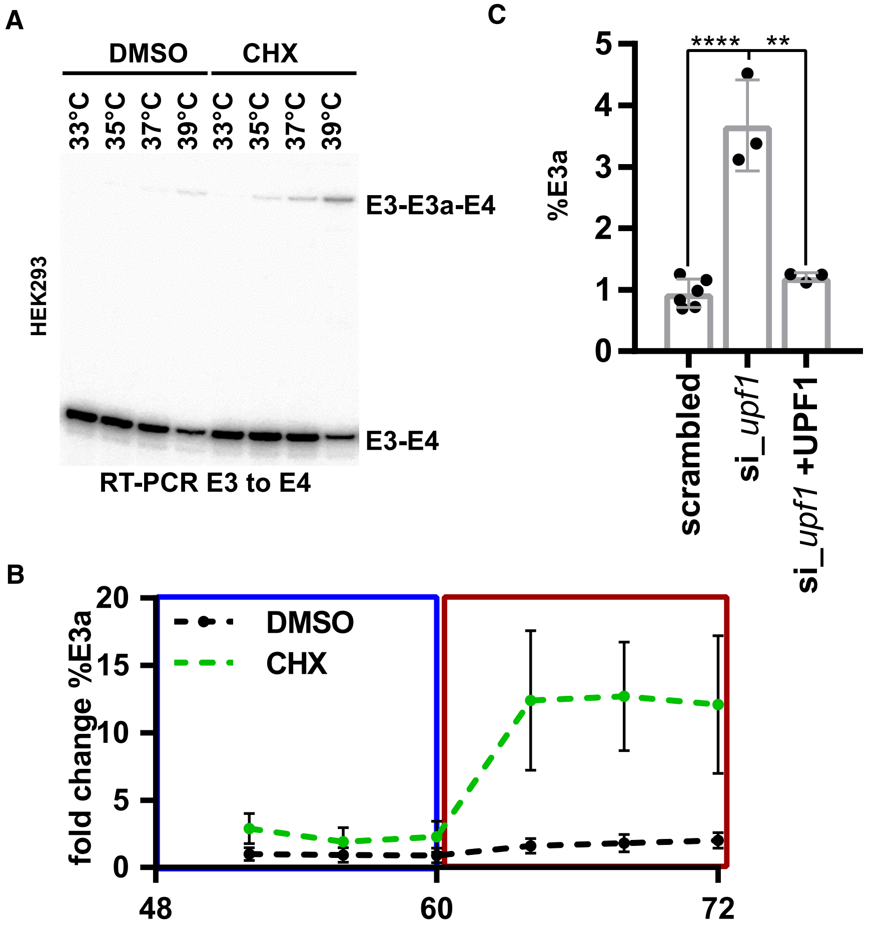

Figure EV1. RBM3 intron 3 contains an evolutionary conserved heat-induced poison exon.

- A Representative gel image showing heat-induced and CHX-stabilized formation of the E3a isoform in human HEK293 cells (quantification in Fig 1C).
- B Rhythmic RBM3 E3a regulation. HEK293 cells were pre-entrained with square-wave temperature cycles (12 h 34°C/12 h 38°C) for 48 h. For the last 24 h, cells were treated with DMSO or CHX every 4 h and harvested after 4 h and analyzed by splicing-sensitive RT–PCR ($n = 6$, mean ± s.d.).
- C RBM3 E3a stabilization in response to UPF1 knockdown and rescue (mean ± s.d., $n = 3$, for scrambled $n = 6$, all individual data points are shown) (Colombo *et al*, 2017). Statistical significance was determined by unpaired *t*-test and is indicated by asterisks: *P*-values: **$P < 0.01$, ****$P < 0.0001$.

© 2023 The Authors

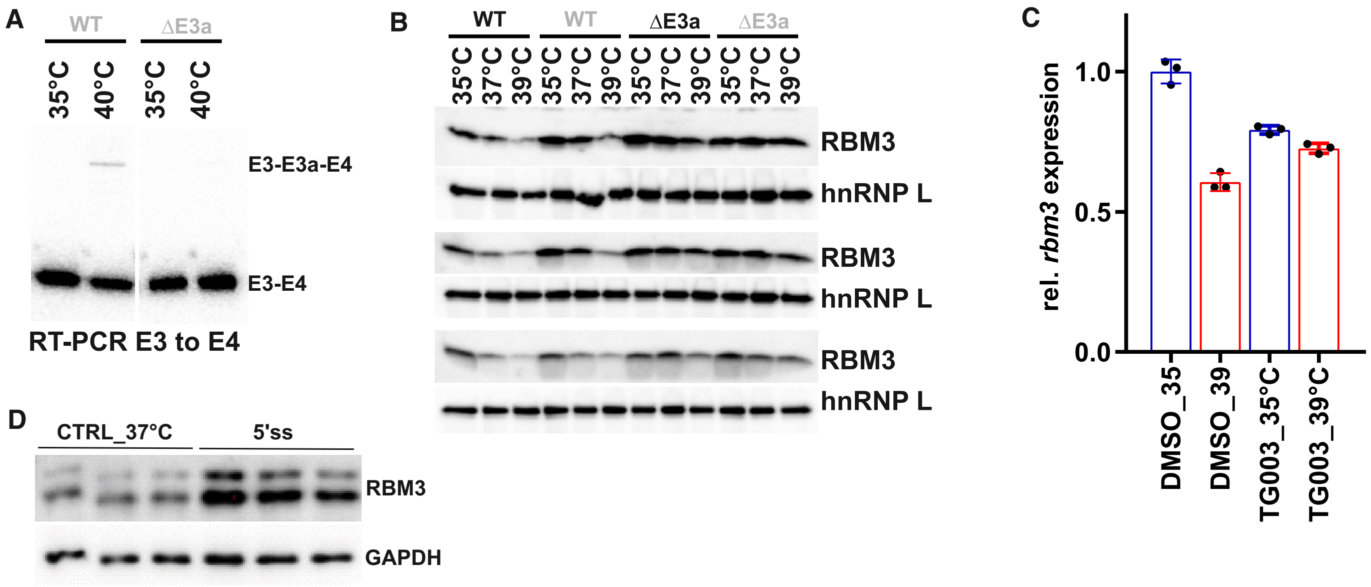

Figure EV2. E3a controls temperature-dependent RBM3 expression.

A An RT–PCR in CHX-treated HEK293 cells confirms CRISPR/CAS9-mediated removal of E3a in a DE3a clone at the RNA level.

B Western blot analysis of RBM3 protein expression, hnRNP L served as a loading control. Two independent WT and ΔE3a clones are shown in biological triplicates. Quantification in Fig 2C.

C Inhibition of CLK1/4 kinase by TG003 abolishes the effect of temperature on *rbm3* expression. Whippet-derived TpM values are shown relative to DMSO 35°C (mean ± s.d., $n = 3$, all individual data points are shown). This reveals an almost twofold difference in *rbm3* levels comparing 6 h DMSO 35 vs. 39°C. Note that this is basically abolished by adding TG003 during the shift from 39 to 35°C (Haltenhof *et al*, 2020).

D Blocking rbm3 E3a inclusion induces RBM3 protein levels. Primary hippocampal neurons were transfected with the indicated MOs for 48 h and investigated by Western blotting. GAPDH served as a loading control. Quantification in Fig 2E.

© 2023 The Authors

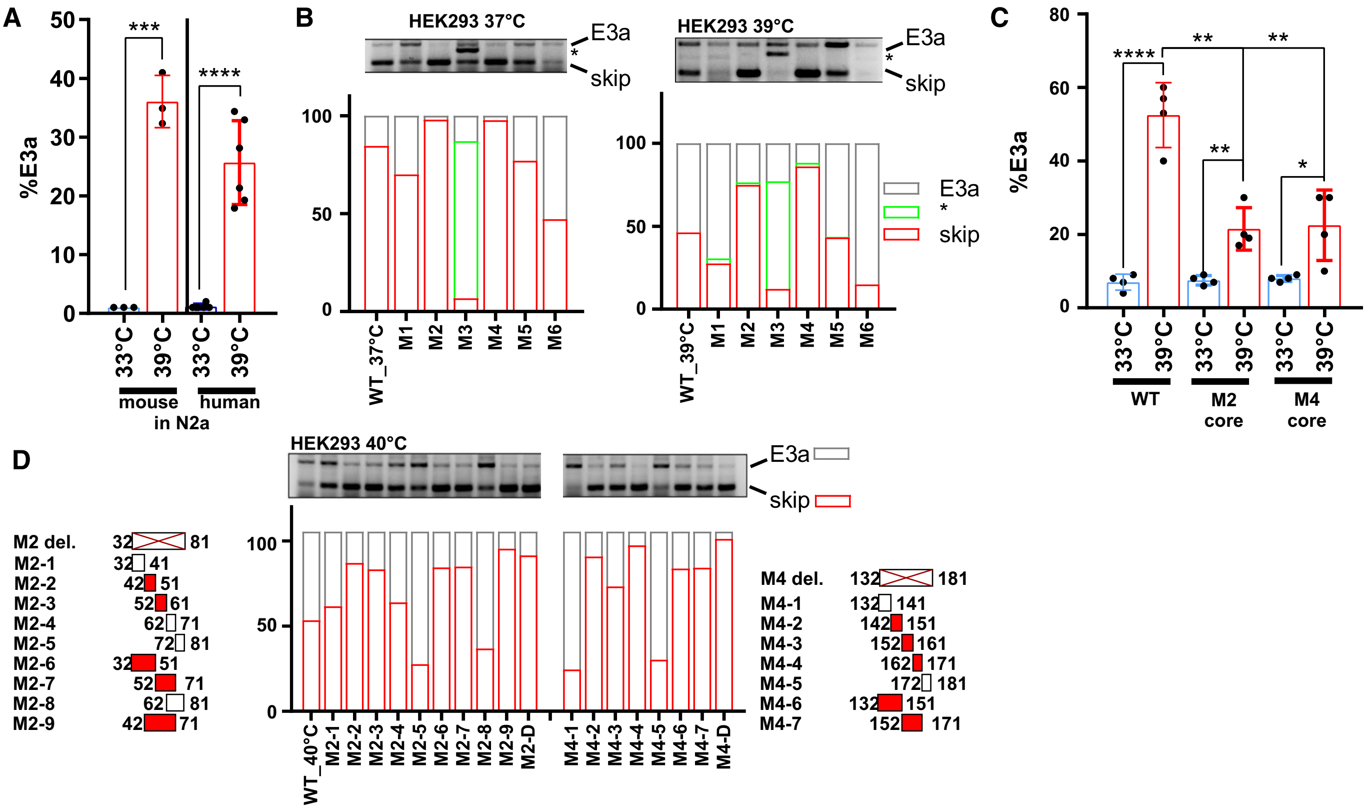

Figure EV3. Mapping cis-regulatory elements controlling RBM3 E3a inclusion.

A An RBM3 minigene reproduces temperature-controlled E3a inclusion. Human and mouse minigenes were transfected into N2a cells and analyzed as in Fig 3A (mean ± s.d., $n = 3$–6, all individual data points are shown).

B Systematic mutational screening for regulatory elements (see Table EV1). The indicated sequences were replaced by sequences from human beta-globin. M2, M4, and M5 contain the same exon 2 sequence from beta-globin exon 2. Only in the M2 and M4 context these sequences prevent inclusion, ruling out the possibility that we included a silencer element. M1 and M3 contain sequences of the beta-globin 3'ss, M6 the beta-globin 5'ss (these mutations do not result in an increased splice site strength). E3a inclusion in HEK293 at 37 and 39°C was investigated by minigene-specific RT–PCR. On top, a representative gel is shown. Below, quantifications of the detected isoforms (mean, $n = 2$). Note that replacing the internal 3'ss with a globin 3'ss promotes its usage.

C Temperature response of the indicated minigenes. We deleted the evolutionary conserved core of the M2 and M4 regions. Briefly, M2-2 and M2-3 are 100% conserved between human and mouse. Thus, M2-2 and M2-3 are regarded as core sequence of the M2 enhancer to be deleted in hRBM3 minigene. For the M4 region, M4-3 is the central region of the conserved sequence. Therefore, M4-3 and a part of the upstream sequence of M4-4 were deleted as core sequence of M4 mutant in the hRBM3 minigene (mean ± s.d., $n = 4$, all individual data points are shown).

D Detailed mutational screening of the M2 region (borders indicated on the left) and M4 region (borders indicated on the right). See Table EV1 for sequences. In M2 or M4 del. The M2 or M4 sequences are removed (and not replaced). In M2-1 to M2-9 and M4-1 to M4-7, the indicated sequences are replaced by human beta-globin exon 2 sequences from the same relative position. On top, a representative PCR image is shown. Below, quantifications of the detected isoforms (mean, $n = 2$). In all panels, statistical significance was determined by unpaired, two-sided t-test and is indicated by asterisks: P-values: $*P < 0.05$, $**P < 0.01$, $***P < 0.001$, $****P < 0.0001$.

© 2023 The Authors

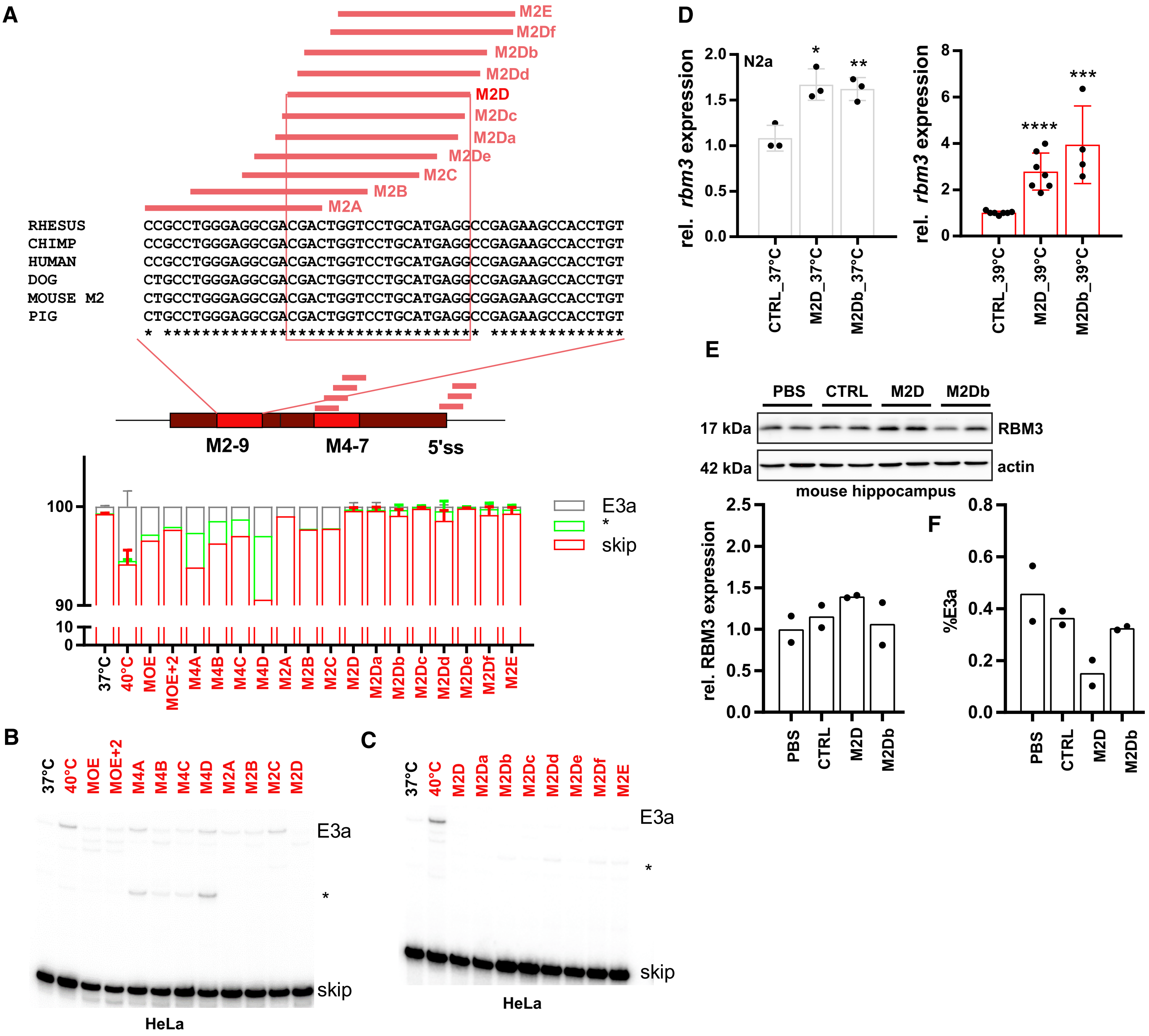

Figure EV4. Screening for ASO sequences that mediate RBM3 E3a exclusion.

A–C ASOs targeting M2-9, M4-7, or the 5'ss (see Table EV2) prevent RBM3 E3a inclusion in human HeLa cells. In (A), ASO-binding sites within the M2, M4, and the 5'ss region are indicated in the middle. On top, for the mouse M2 region an alignment of mouse, human, chimp, rhesus, dog, and pig sequences is shown. ASO-binding sites are indicated, and the 100% conserved binding site of M2D is boxed. ASOs were transfected and cells were kept at 40°C for 24 h. Control samples at 37 and 40°C are shown. All samples were treated with CHX for the last 4 h. Exon 3a inclusion was investigated by splicing-sensitive radioactive RT–PCR. In (A) (bottom), a quantification is shown (mean ± s.d., $n = 2$; $n = 5$ for all M2D variants and M2E, $n = 1$ for M4B). In (B) and (C) representative gels are shown. The asterisk marks the use of internal 5′ and 3'ss that is promoted by all ASOs targeting the M4 region. Note that all variants targeting the M2D region almost quantitatively prevent E3a inclusion and, at 40°C, lead to inclusion levels that are lower than the one observed at 37°C for control cells.

D M2D and M2Db induce *rbm3* mRNA expression in mouse N2a cells. ASOs were transfected for 24 h at 37°C (left) or at 39°C (right). rbm3 induction was measured relative to a CTRL ASO and relative to HPRT expression (mean ± s.d., $n \geq 3$, all individual data points are shown; unpaired *t*-test derived *P*-value *$P < 0.5$, **$P < 0.01$, ***$P < 0.001$, ****$P < 0.0001$).

E M2D, but not M2Db, induces RBM3 protein expression *in vivo* (100 µg dose per mouse). Hippocampus samples from two independent mice per condition were analyzed by Western blotting (top), and RBM3 signal was quantified relative to Actin and PBS (bottom, mean ± s.d., $n = 2$).

F M2D, but not M2Db, reduces E3a inclusion *in vivo* (300 µg dose per mouse). Cerebellum RNA samples from two independent mice per condition were analyzed by splicing-sensitive RT–PCR, and %E3a signal was quantified (mean ± s.d., $n = 2$).

 © 2023 The Authors
