# Supplementary figures and images for: ASO targeting RBM3 temperature‐controlled poison exon splicing prevents neurodegeneration in vivo
Source: EMBO Mol Med. 2023 Mar 22;15(5):e17157. doi: 10.15252/emmm.202217157 (PMC10165353; doi:10.15252/emmm.202217157)

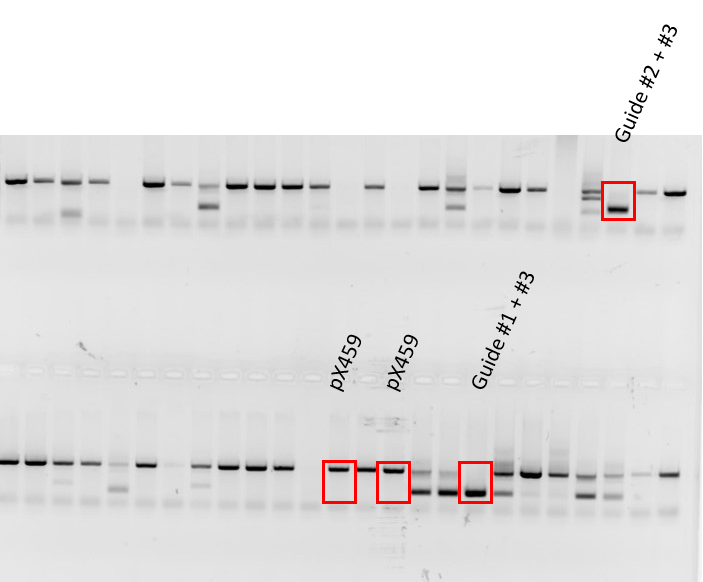

Supplement: Supplementary file 6 — Source Data for Figure 2 [file EMMM-15-e17157-s001.zip › Figure 2/2A/manuscriptEMM-2022-17157_SourceDataForFigure2A.tif]

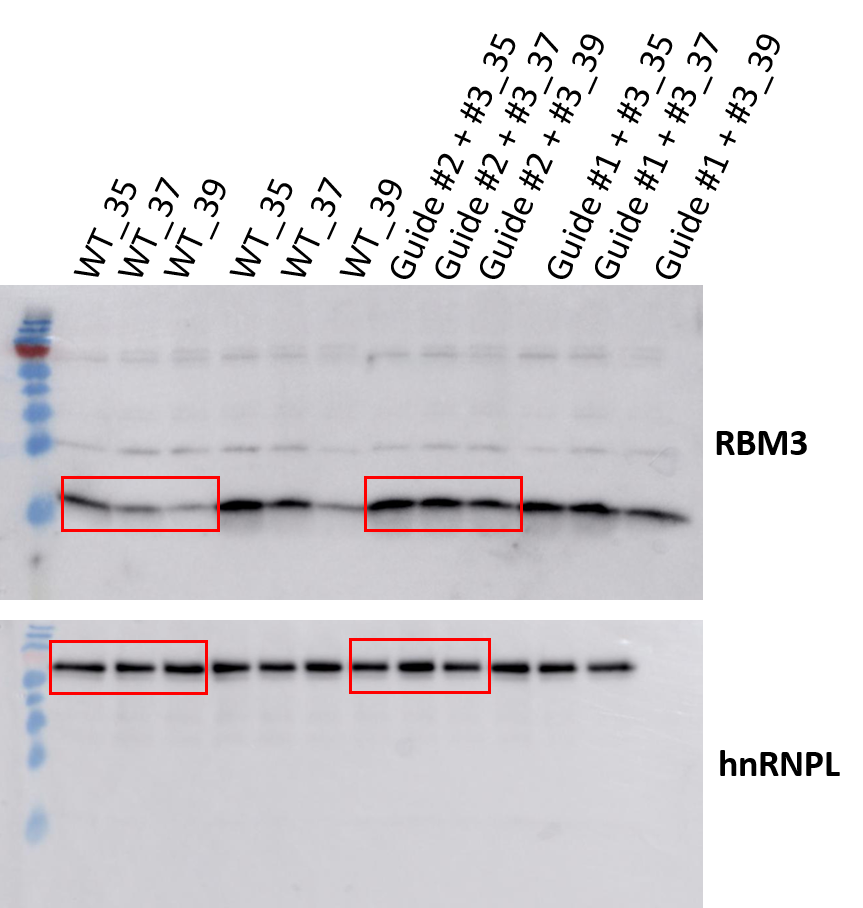

Supplement: Supplementary file 6 — Source Data for Figure 2 [file EMMM-15-e17157-s001.zip › Figure 2/2C/manuscriptEMM-2022-17157_SourceDataForFigure2C.tif]

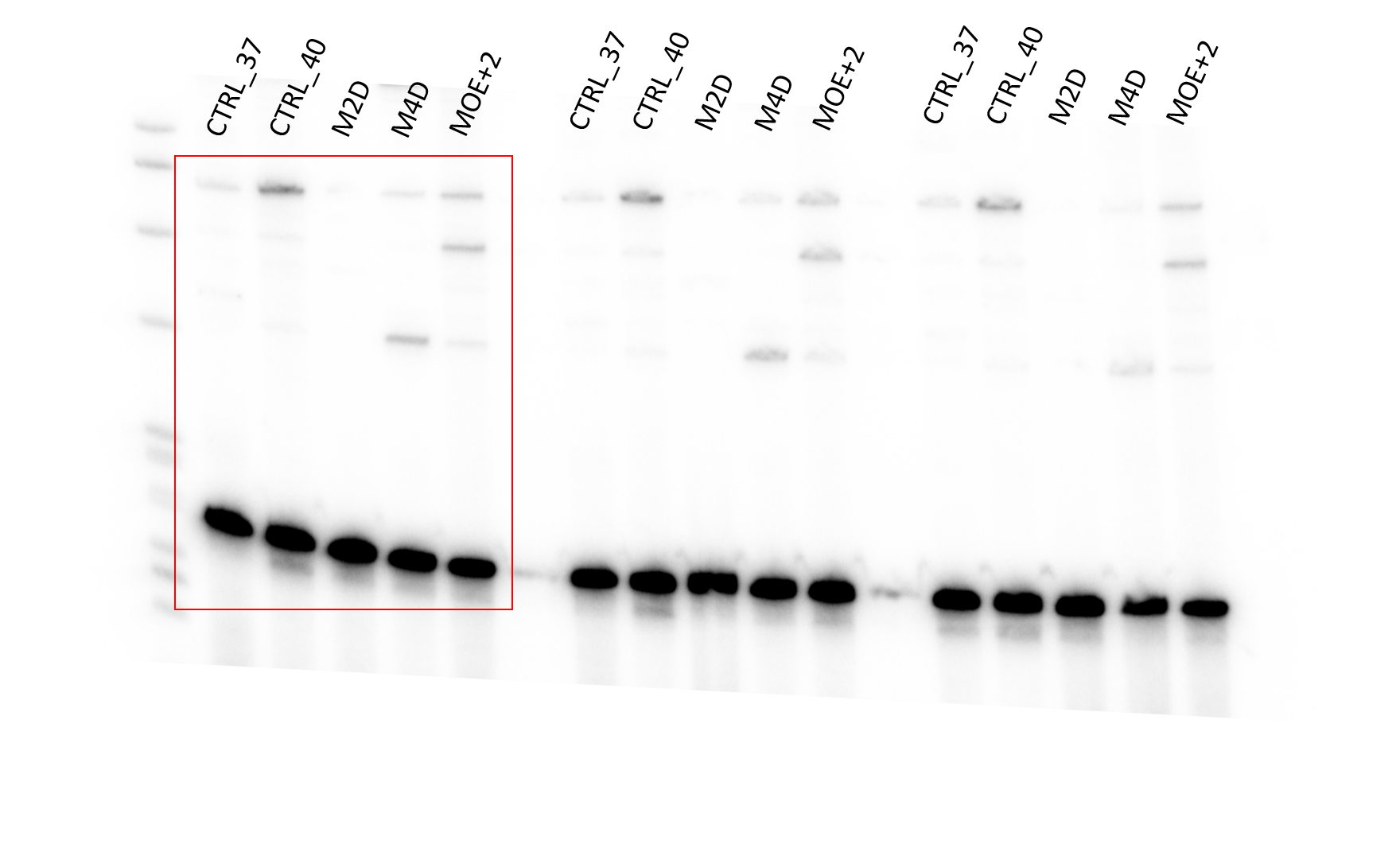

Supplement: Supplementary file 7 — Source Data for Figure 3 [file EMMM-15-e17157-s004.zip › Figure 3/3D/manuscriptEMM-2022-17157_SourceDataForFigure3D.tif]

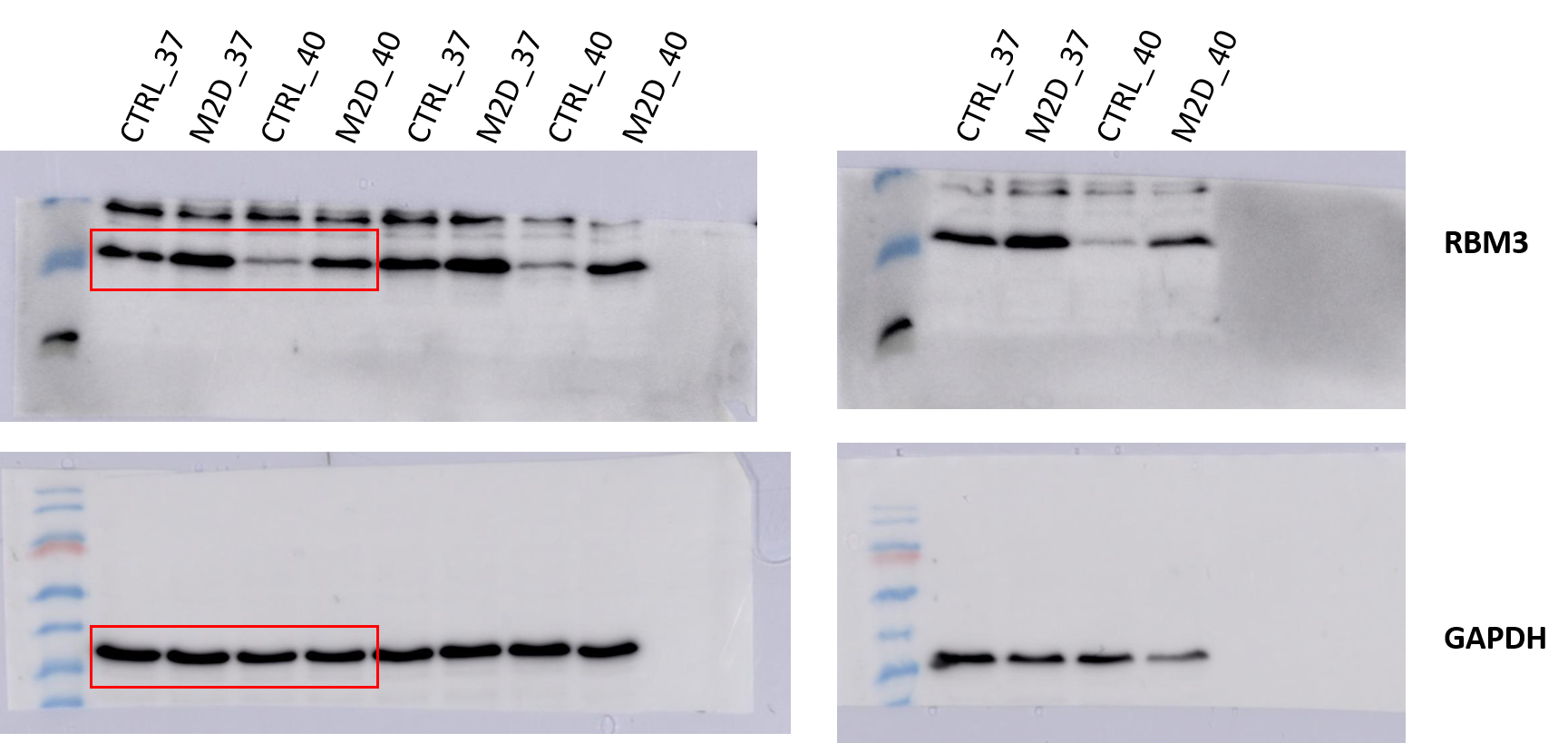

Supplement: Supplementary file 7 — Source Data for Figure 3 [file EMMM-15-e17157-s004.zip › Figure 3/3F/manuscriptEMM-2022-17157_SourceDataForFigure3F.tif]

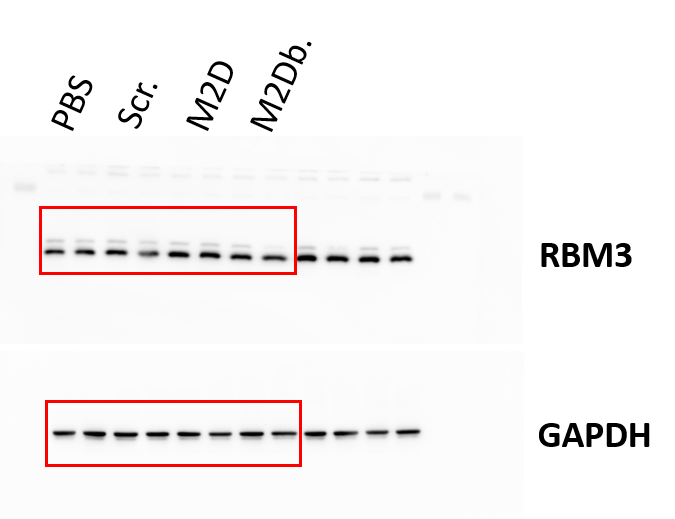

Supplement: Supplementary file 7 — Source Data for Figure 3 [file EMMM-15-e17157-s004.zip › Figure 3/3G/manuscriptEMM-2022-17157_SourceDataForFigure3G.tif]

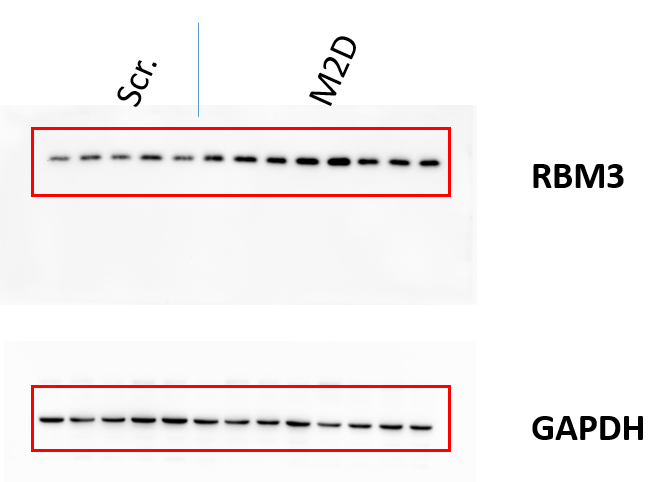

Supplement: Supplementary file 8 — Source Data for Figure 4 [file EMMM-15-e17157-s008.zip › Figure 4/4B/manuscriptEMM-2022-17157_SourceDataForFigure4B.tif]

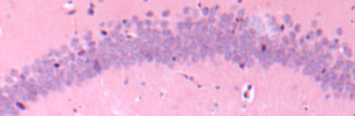

Supplement: Supplementary file 8 — Source Data for Figure 4 [file EMMM-15-e17157-s008.zip › Figure 4/4C/manuscriptEMM-2022-17157_SourceDataForFigure4C_NBH 4X crop zoom.tif]

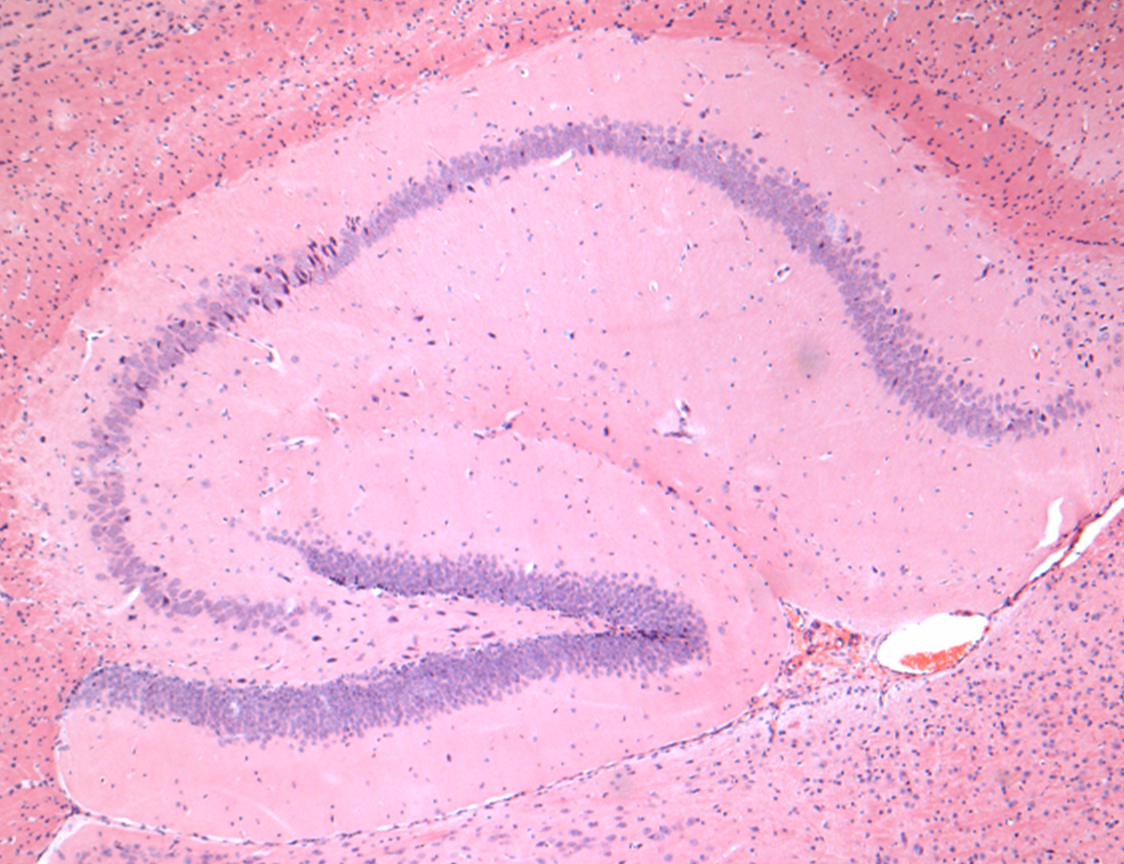

Supplement: Supplementary file 8 — Source Data for Figure 4 [file EMMM-15-e17157-s008.zip › Figure 4/4C/manuscriptEMM-2022-17157_SourceDataForFigure4C_NBH 4X crop.tif]

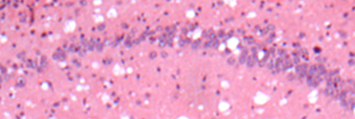

Supplement: Supplementary file 8 — Source Data for Figure 4 [file EMMM-15-e17157-s008.zip › Figure 4/4C/manuscriptEMM-2022-17157_SourceDataForFigure4C_prion + control ASO 4X crop zoom.tif]

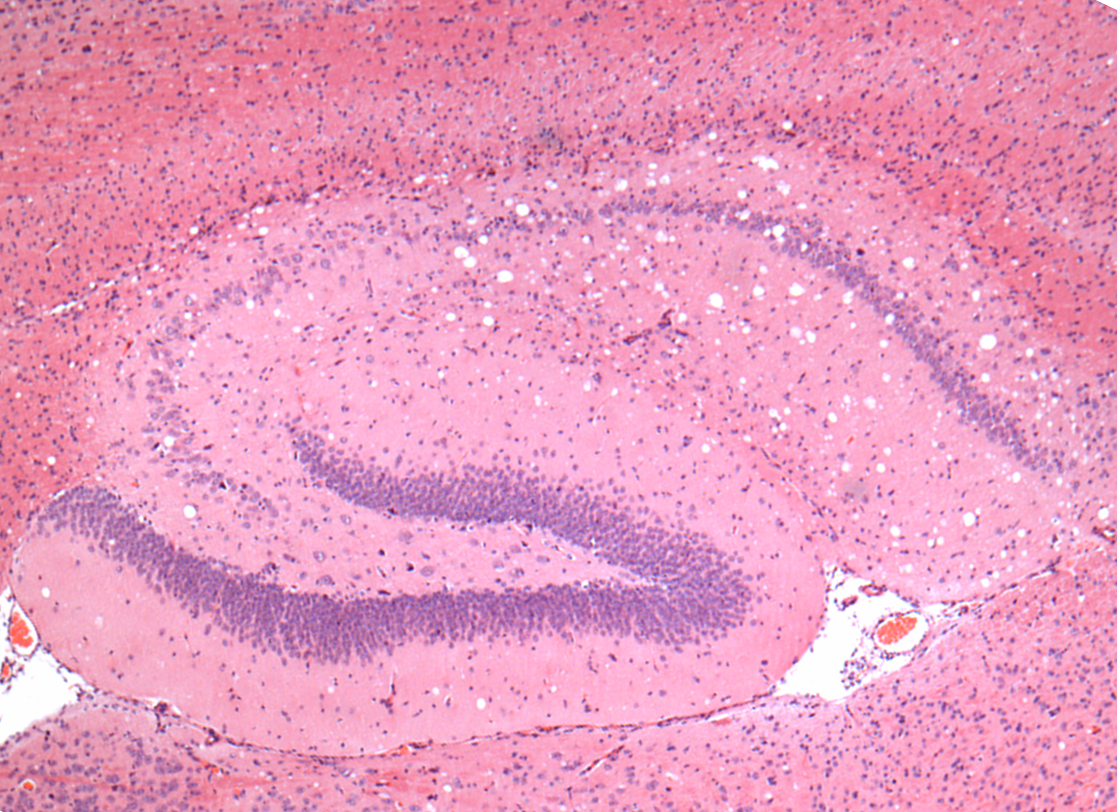

Supplement: Supplementary file 8 — Source Data for Figure 4 [file EMMM-15-e17157-s008.zip › Figure 4/4C/manuscriptEMM-2022-17157_SourceDataForFigure4C_prion + control ASO 4X crop.tif]

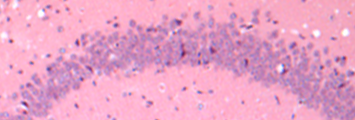

Supplement: Supplementary file 8 — Source Data for Figure 4 [file EMMM-15-e17157-s008.zip › Figure 4/4C/manuscriptEMM-2022-17157_SourceDataForFigure4C_prion + M2D ASO 4X crop zoom.tif]

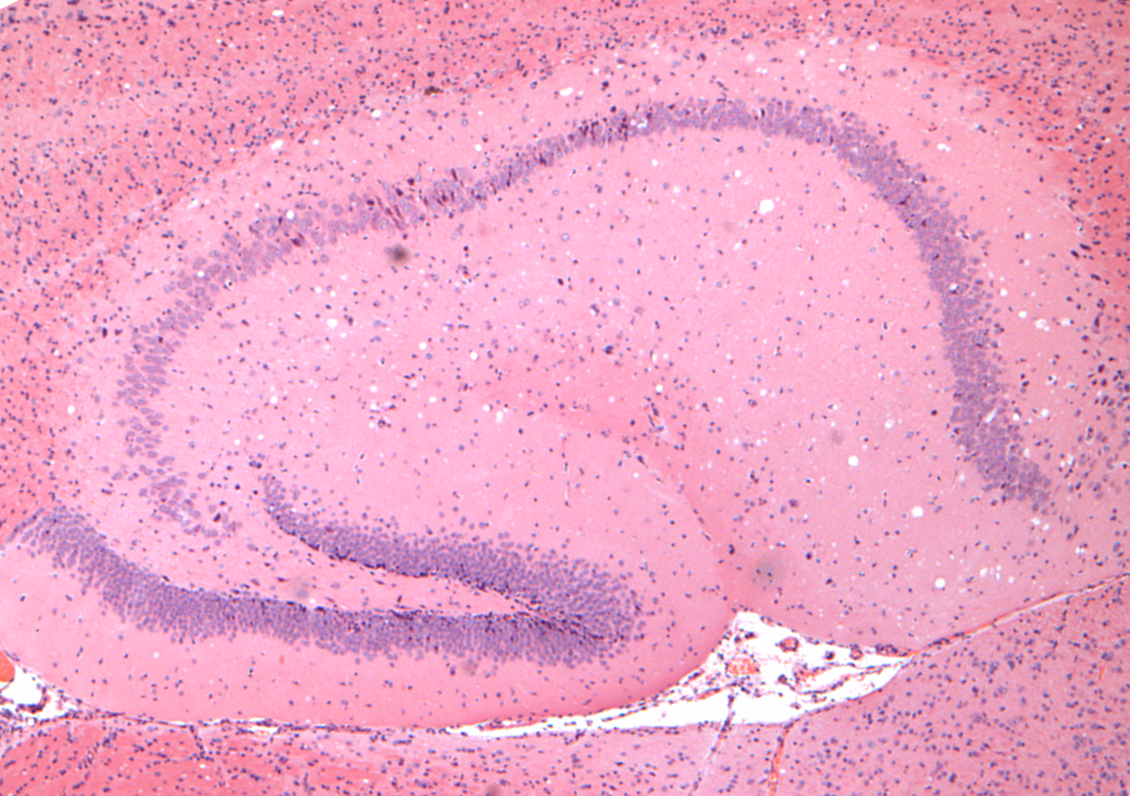

Supplement: Supplementary file 8 — Source Data for Figure 4 [file EMMM-15-e17157-s008.zip › Figure 4/4C/manuscriptEMM-2022-17157_SourceDataForFigure4C_prion + M2D ASO 4X crop.tif]

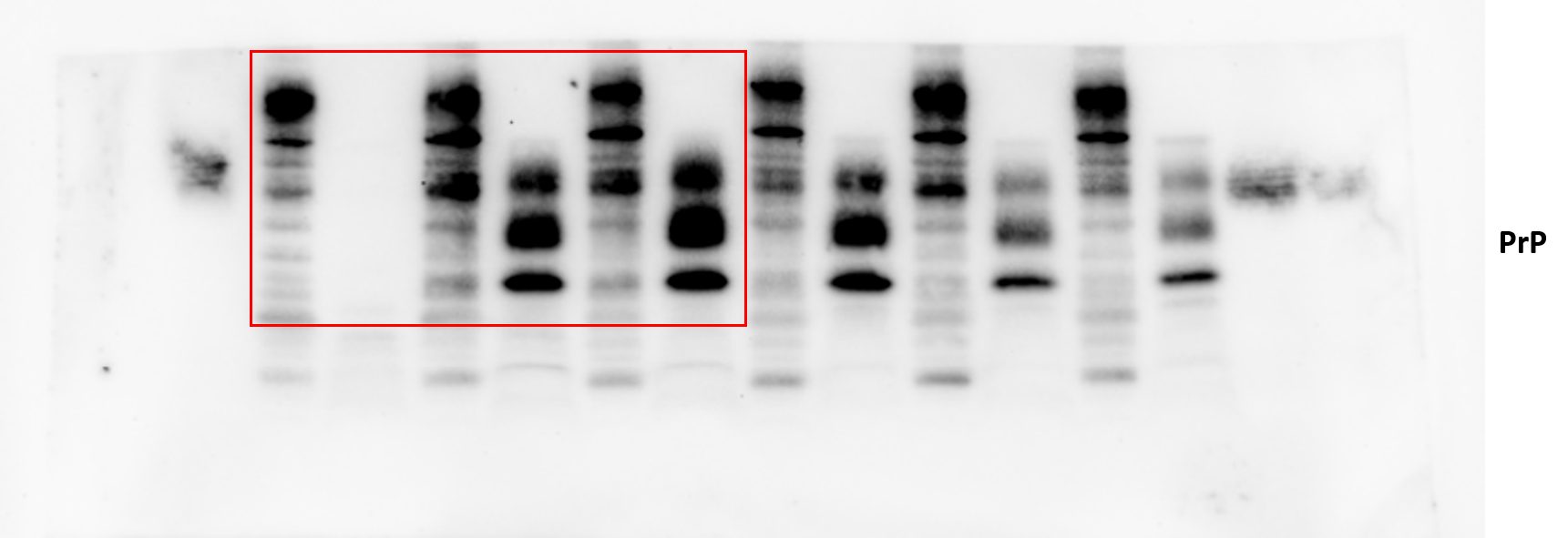

Supplement: Supplementary file 8 — Source Data for Figure 4 [file EMMM-15-e17157-s008.zip › Figure 4/4F/manuscriptEMM-2022-17157_SourceDataForFigure4F.png]
