# Supplementary material for: ASO targeting RBM3 temperature‐controlled poison exon splicing prevents neurodegeneration in vivo
Source: EMBO Mol Med. 2023 Mar 22;15(5):e17157. doi: 10.15252/emmm.202217157 (PMC10165353; doi:10.15252/emmm.202217157)
Supplement: Supplementary file 9 [file EMMM-15-e17157-s007.pdf]

EMBO Molecular Medicine

ASO targeting temperature-controlled RBM3 poison exon splicing prevents neurodegeneration in vivo

Marco Preußner, Heather Smith, Daniel Hughes, Min Zhang, Ann-Kathrin Emmerichs, Silvia Scalzitti, Diego Peretti, Dean Swinden, Alexander Neumann, Tom Halteenhof, Giovanna Mallucci, and Florian Heyd
DOI: 10.15252/emmm.202217157

Corresponding authors: Florian Heyd (florian.heyd@fu-berlin.de) , Marco Preußner (mpreussner@zedat.fu-berlin.de), Giovanna Mallucci (gmallucci@altoslabs.com)

Review Timeline:

Submission Date:	9th Nov 22
Editorial Decision:	29th Dec 22
Revision Received:	16th Jan 23
Editorial Decision:	10th Feb 23
Revision Received:	16th Feb 23
Accepted:	18th Feb 23

Editor: Kelly Anderson

Transaction Report:

(Note: With the exception of the correction of typographical or spelling errors that could be a source of ambiguity, letters and reports are not edited. Depending on transfer agreements, referee reports obtained elsewhere may or may not be included in this compilation. Referee reports are anonymous unless the Referee chooses to sign their reports.)

29th Dec 2022

Dear Dr. Heyd,

Thank you for the submission of your manuscript to EMBO Molecular Medicine. We have now received feedback from the reviewers who agreed to evaluate your manuscript. As you will see from the reports below, the referees acknowledge the interest of the study and are overall supporting publication of your work pending appropriate revisions.

Addressing the reviewers' concerns in full will be necessary for further consideration of the manuscript in our journal, and acceptance of the manuscript will entail a second round of review. EMBO Molecular Medicine encourages a single round of revision only and therefore, acceptance or rejection of the manuscript will depend on the completeness of your responses included in the next, final version of the manuscript. For this reason, and to save you from any frustrations in the end, I would strongly advise against returning an incomplete revision. It would be good to discuss your plan to address referee concerns and I am available to do so via zoom or email in the new few weeks.

Revised manuscripts should be submitted within three months of a request for revision; they will otherwise be treated as new submissions, except under exceptional circumstances in which a short extension is obtained from the editor.

I look forward to seeing a revised form of your manuscript as soon as possible.

Yours sincerely,

Kelly

Kelly M Anderson, PhD
Scientific Editor
EMBO Molecular Medicine

*****

When submitting your revised manuscript, please carefully review the instructions that follow below. We perform an initial quality control of all revised manuscripts before re-review; failure to include requested items will delay the evaluation of your revision.

We require:

1) A .docx formatted version of the manuscript text (including legends for main figures, EV figures and tables). Please make sure that the changes are highlighted to be clearly visible.

2) Individual production quality figure files as .eps, .tif, .jpg (one file per figure). For guidance, download the 'Figure Guide PDF': (https://www.embopress.org/page/journal/17574684/authorguide#figureformat).

3) A .docx formatted letter INCLUDING the reviewers' reports and your detailed point-by-point responses to their comments. As part of the EMBO Press transparent editorial process, the point-by-point response is part of the Review Process File (RPF), which will be published alongside your paper.

4) A complete author checklist, which you can download from our author guidelines (https://www.embopress.org/page/journal/17574684/authorguide#submissionofrevisions). Please insert information in the checklist that is also reflected in the manuscript. The completed author checklist will also be part of the RPF.

5) Please note that all corresponding authors are required to supply an ORCID ID for their name upon submission of a revised manuscript.

6) It is mandatory to include a 'Data Availability' section after the Materials and Methods. Before submitting your revision, primary datasets produced in this study need to be deposited in an appropriate public database, and the accession numbers and database listed under 'Data Availability'. Please remember to provide a reviewer password if the datasets are not yet public (see https://www.embopress.org/page/journal/17574684/authorguide#dataavailability).

In case you have no data that requires deposition in a public database, please state so in this section. Note that the Data Availability Section is restricted to new primary data that are part of this study.

7) For data quantification: please specify the name of the statistical test used to generate error bars and P values, the number (n) of independent experiments (specify technical or biological replicates) underlying each data point and the test used to calculate p-values in each figure legend. The figure legends should contain a basic description of n, P and the test applied. Graphs must include a description of the bars and the error bars (s.d., s.e.m.). See also 'Figure Legend' guidelines: https://www.embopress.org/page/journal/17574684/authorguide#figureformat

8) At EMBO Press we ask authors to provide source data for the main manuscript figures. Our source data coordinator will contact you to discuss which figure panels we would need source data for and will also provide you with helpful tips on how to upload and organize the files.

9) Our journal encourages inclusion of *data citations in the reference list* to directly cite datasets that were re-used and obtained from public databases. Data citations in the article text are distinct from normal bibliographical citations and should directly link to the database records from which the data can be accessed. In the main text, data citations are formatted as follows: "Data ref: Smith et al, 2001" or "Data ref: NCBI Sequence Read Archive PRJNA342805, 2017". In the Reference list, data citations must be labeled with "[DATASET]". A data reference must provide the database name, accession number/identifiers and a resolvable link to the landing page from which the data can be accessed at the end of the reference. Further instructions are available at .

10) We replaced Supplementary Information with Expanded View (EV) Figures and Tables that are collapsible/expandable online. A maximum of 5 EV Figures can be typeset. EV Figures should be cited as 'Figure EV1, Figure EV2" etc... in the text and their respective legends should be included in the main text after the legends of regular figures.

- For the figures that you do NOT wish to display as Expanded View figures, they should be bundled together with their legends in a single PDF file called *Appendix*, which should start with a short Table of Content. Appendix figures should be referred to in the main text as: "Appendix Figure S1, Appendix Figure S2" etc.

- Additional Tables/Datasets should be labeled and referred to as Table EV1, Dataset EV1, etc. Legends have to be provided in a separate tab in case of .xls files. Alternatively, the legend can be supplied as a separate text file (README) and zipped together with the Table/Dataset file.

See detailed instructions here:

.

11) The paper explained: EMBO Molecular Medicine articles are accompanied by a summary of the articles to emphasize the major findings in the paper and their medical implications for the non-specialist reader. Please provide a draft summary of your article highlighting

- the medical issue you are addressing,

- the results obtained and

- their clinical impact.

This may be edited to ensure that readers understand the significance and context of the research. Please refer to any of our published articles for an example.

12) For more information: There is space at the end of each article to list relevant web links for further consultation by our readers. Could you identify some relevant ones and provide such information as well? Some examples are patient associations, relevant databases, OMIM/proteins/genes links, author's websites, etc...

13) Author contributions: You will be asked to provide CRediT (Contributor Role Taxonomy) terms in the submission system. These replace a narrative author contribution section in the manuscript.

14) A Conflict of Interest statement should be provided in the main text.

15) Every published paper now includes a 'Synopsis' to further enhance discoverability. Synopses are displayed on the journal webpage and are freely accessible to all readers. They include a short stand first (maximum of 300 characters, including space) as well as 2-5 one-sentences bullet points that summarizes the paper. Please write the bullet points to summarize the key NEW findings. They should be designed to be complementary to the abstract - i.e. not repeat the same text. We encourage inclusion of key acronyms and quantitative information (maximum of 30 words / bullet point). Please use the passive voice. Please attach these in a separate file or send them by email, we will incorporate them accordingly.

Please also suggest a striking image or visual abstract to illustrate your article as a PNG file 550 px wide x 300-600 px high.

EMBO Molecular Medicine has a "scooping protection" policy, whereby similar findings that are published by others during review or revision are not a criterion for rejection. Should you decide to submit a revised version, I do ask that you get in touch after three months if you have not completed it, to update us on the status.

Please note: When submitting your revision you will be prompted to enter your funding and payment information. This will allow Wiley to send you a quote for the article processing charge (APC) in case of acceptance. This quote takes into account any reduction or fee waivers that you may be eligible for. Authors do not need to pay any fees before their manuscript is accepted and transferred to the publisher.

EMBO Press participates in many Publish and Read agreements that allow authors to publish Open Access with reduced/no publication charges. Check your eligibility: https://authorservices.wiley.com/author-resources/Journal-Authors/open-access/affiliation-policies-payments/index.html

***** Reviewer's comments *****

Referee #2 (Comments on Novelty/Model System for Author):

Well the Novelty and medical impact would be improved by knowing you could get good distribution in a larger animal and that no tox arose.

Referee #2 (Remarks for Author):

The paper by Preußner et al. describes the use of an ASO to alter the amount of RBM3 produced the MOE chemistry is used which is the same as that used in the FDA approved drug Spinraza used to treat SMA. The RBM3 is a gene that is associated with a response to cold shock and acts as a neuroprotectant it is also indicated to increase cell proliferation and has been implicated as a protooncogene. The ASO used is conserved in humans and mice which is useful for translation (Namely M2D). The authors do show effectiveness in the Prion model and as such this is useful information. The main factor that should be toned down while this certainly is an interesting target neuroprotection has a relatively poor record in translation so at the moment it is relatively unclear how well this will translate for neurodegenerative disorders. The authors for instance only give the positive expel of spinraza and do not discus the negative ASO trials in Huntington's. My specific comment are as follows

1) Doses of 100ug to 300ug of ASO seem relatively high compared to SMA treatments this does get somewhat confusing as the mice in SMA were tested at 20ug/g when given through the CSF whereas the clinical dose in patients is 12 mg so an infant of 3000g so .012g/ 3000g which is 4ug/ g. It just should be clear how the doses compare you can usually also see a dose response. The amount given to the mouse here is 300ug assuming a weight of 23grams this is 13ug/g. So, in a similar range to what was given to the mouse but lower than the dose used in humans.

2) The authors use w.p.i for post inoculation is a little confusing as it could also be post injection it might help to clarify that all time points are relative to inoculation with Prion.

3) The RBM3 levels could change with Prion infection as it is a stress can a comparison between a Prion infected and non-infected animal be given so as to ensure the level of change is not just due to infection I presume not as the untreated Prion infected animals are a comparison group.

4) The authors indicate an overly positive view on the potential of RMB3 for treatment of a series. diverse conditions, from acute treatment of neonates through to cardiac surgery, stroke and head injury in adults, to longer term neuroprotection in degenerative disorders. First it is not really clear in most of these situations how much protecting a neuron is going to do if it is alive and not functioning. Second neuroprotection has not as yet really proved very effective in the clinic. So yes it is what is wanted but we need to waith to really see this is the case. In particular the authors should discuss the negative results in ASO clinical trials as well as the positive. In the case of Huntingtons the ASOs to knockdown Huntingtons appeared to work in mice but failed in clinical trials. The inatial trials of ASOs in myotonic also failed most likely due to inefficient uptake by muscle. Both these programs used MOEs. Often the exact reason for failure can be difficult to determine but the positive of SMA should be balanced with some of the negatives in discussion. It is also likely that large animal studies of the ASO will need to be done so as to ensure safety and no adverse effects or cancer induction. The licensing of the ASOs in ALS does not mean there are no problems ahead to overcome. The discussion needs to be more balanced.

5) The ASO sequences are given in a supplementary table but the complete sequence of Exon 3b could be given in the figures with a bar above indicating the critical oligos in the case of the ASOs a numbering system from the intron was used this form of naming could also be added to the M2 name ie M2 32-47 (this is not the correct number for M2 ASO just an example of the numbering system) ie the exon base number from the 5 prime boundary this will be useful in reading the text. It is also quite lucky that the sequence of M2 is conserved and would be useful to know if it is conserved in all larger species that could be used

for tox ie pig dog and monkey.

Referee #3 (Comments on Novelty/Model System for Author):

This is a smart study, very well described, from an initial phase of ASO design, followed by an in vitro analysis and validation; and with a final in vivo testing. The authors have also a strong background the in the study of RBM3 that supports the results and the final conclusion of the analysis.

Referee #3 (Remarks for Author):

The article submitted by Marco Preußner et al to EMBO Molecular Medicine journal, and titled "ASO targeting temperature-controlled RBM3 poison exon splicing prevents neurodegeneration in vivo" describes the potential utility of the ASO therapeutic strategy to induce the increase of RBM3 expression, without the natural RBM3- inductor; cooling.
The authors report that a single administration of ASO to exclude the poison exon, results in long-lasting increase of RBM3 expression in cells and mouse brains. In addition, in prion-diseased mice, this treatment leads to remarkable neuroprotection, with prevention of neuronal loss. The authors conclude that this novel approach used to stimulate the expression of RBM3 open the possibility to explore the beneficial effect of this cold-dependent protein to induce brain protection in acute brain injury or Alzheimer's disease, by avoiding the sides effect of hypothermia.
This is a smart study, very well described, from an initial phase of ASO design, followed by an in vitro analysis and validation; and with a final in vivo testing. The authors have also a strong background the in the study of RBM3 that supports the results and the final conclusion of the analysis.
Chemical agents or agonists to induce the pharmacological expression of RBM3 have been also suggested in other articles without good results so far, and this new approach is a smart alternative strategy to explore the therapeutic use of RBM3.

Some minor points related with the future application of ASO-RBM3 could be included in the discussion of the manuscript.

A recent study published in stroke (J Clin Med. 2022 Feb 11;11(4):949) and supported by previous studies (J. Cereb. Blood Flow Metab. 2019;39:2355-2367; Neuroscience. 2015;305:268-278.), suggests that the recombinant form of the hormone FGF21 could be used as an external therapy to modulate the expression of RBM3. This strategy seems more translational than ASO therapy.

The use of ASO has been tested in experimental animals as a single intracerebroventricular injection that is no so invasive, but thinking in the potential application in old patients with Alzheimer or stroke (for instance), alternative and less invasive routes of administration are more convenient.

Based on the previous study published by same authors (Life Sci Alliance. 2021 Feb 9;4(4):e202000884); Was TrkB signalling analysed to guarantee that the protective effect was mediated by RBM3?

ASO targeting temperature-controlled RBM3 poison exon splicing prevents neurodegeneration in vivo

Response to Referees

We thanks the Reviewers for their constructive and insightful comments. The Referees' comments are reproduced in full. Our responses follow each point in blue text.

Referee #2 (Comments on Novelty/Model System for Author):

Well the Novelty and medical impact would be improved by knowing you could get good distribution in a larger animal and that no tox arose.

We agree with the Referee and larger animal studies will follow this one. We mention this is the discussion (p7). We saw no signs of toxicity for ASO M2D in mice up to 9 weeks after inoculation (throughout the duration of the experiment). We have added a line to this effect on p6.

Referee #2 (Remarks for Author):

The paper by Preußner et al. describes the use of an ASO to alter the amount of RBM3 produced the MOE chemistry is used which is the same as that used in the FDA approved drug Spinraza used to treat SMA. The RBM3 is a gene that is associated with a response to cold shock and acts as a neuroprotectant it is also indicated to increase cell proliferation and has been implicated as a protooncogene. The ASO used is conserved in humans and mice which is useful for translation (Namely M2D). The authors do show effectiveness in the Prion model and as such this is useful information. The main factor that should be toned down while this certainly is an interesting target neuroprotection has a relatively poor record in translation so at the moment it is relatively unclear how well this will translate for neurodegenerative disorders. The authors for instance only give the positive expel of spinraza and do not discus the negative ASO trials in Huntington's. My specific comment are as follows

1) Doses of 100ug to 300ug of ASO seem relatively high compared to SMA treatments this does get somewhat confusing as the mice in SMA were tested at 20ug/g when given through the CSF whereas the clinical dose in patients is 12 mg so an infant of 3000g so .012g/ 3000g which is 4ug/ g. It just should be clear how the doses compare you can usually also see a dose response. The amount given to the mouse here is 300ug assuming a weight of 23grams this is 13ug/g. So, in a similar range to what was given to the mouse but lower than the dose used in humans.

We agree. In fact, for the prion study we used 200ug, which is around 8ug/g. This remains within the range for application in humans cited by the reviewer. We have clarified this in the text on p6.

2) The authors use w.p.i for post inoculation is a little confusing as it could also be post injection it might help to clarify that all time points are relative to inoculation with Prion.

We have made this clear in the text on p6.

3) The RBM3 levels could change with Prion infection as it is a stress can a comparison between a Prion infected and non-infected animal be given so as to ensure the level of change is not just due to infection I presume not as the untreated Prion infected animals are a comparison group.

RBM3 shows non-statistically significant increase in prion infection (see Peretti et al, 2015, *Nature* 518: 236-239), but the key control here is the effect on RBM3 of the ASO M2D vs control ASO in disease. In addition, control ASOs do not increase RBM3 levels in wild type mice (see Fig. 3G).

4) The authors indicate an overly positive view on the potential of RMB3 for treatment of a series. diverse conditions, from acute treatment of neonates through to cardiac surgery, stroke and head injury in adults, to longer term neuroprotection in degenerative disorders. First it is not really clear in most of these situations how much protecting a neuron is going to do if it is alive and not functioning. Second neuroprotection has not as yet really proved very effective in the clinic. So yes it is what is wanted but we need to waith to really see this is the case. In particular the authors should discuss the negative results in ASO clinical trials as well as the positive. In the case of Huntingtons the ASOs to knockdown Huntingtons appeared to work in mice but failed in clinical trials. The inatial trials of ASOs in myotonic also failed most likely due to inefficient uptake by muscle. Both these programs used MOEs. Often the exact reason for failure can be difficult to determine but the positive of SMA should be balanced with some of the negatives in discussion. It is also likely that large animal studies of the ASO will need to be done so as to ensure safety and no adverse effects or cancer induction. The licensing of the ASOs in ALS does not mean there are no problems ahead to overcome. The discussion needs to be more balanced.

We thank the Referee for bringing up these points. We have addressed these comments for a more balanced discussion on p7 and specifically mention the Huntingtons trial and the need for additional studies in larger animals.

5) The ASO sequences are given in a supplementary table but the complete sequence of Exon 3b could be given in the figures with a bar above indicating the critical oligos in the case of the ASOs a numbering system from the intron was used this form of naming could also be added to the M2 name ie M2 32-47 (this is not the correct number for M2 ASO just an example of the numbering system) ie the exon base number from the 5 prime boundary this will be useful in reading the text. It is also quite lucky that the sequence of M2 is conserved and would be useful to know if it is conserved in all larger species that could be used for tox ie pig dog and monkey.

Sequences for human and mouse E3a are too large to present them within one clear Figure, so we provide these sequences in Table S1. As we would prefer to stick to our nomenclature, the exact binding positions within exon 3a are now provided in Table S2. Additionally, we provide an alignment of human, chimp, rhesus, dog, pig and mouse M2 sequences (in Figure EV4A) and highlight the ASO binding sites within these alignments. This reveals 100% conservation for the M2D target site across all these species.

Referee #3 (Comments on Novelty/Model System for Author):

This is a smart study, very well described, from an initial phase of ASO design, followed by an in vitro analysis and validation; and with a final in vivo testing. The authors have also a strong background the in the study of RBM3 that supports the results and the final conclusion of the analysis.

Referee #3 (Remarks for Author):

The article submitted by Marco Preußner et al to EMBO Molecular Medicine journal, and titled "ASO targeting temperature-controlled RBM3 poison exon splicing prevents neurodegeneration in vivo" describes the potential utility of the ASO therapeutic strategy to induce the increase of RBM3 expression, without the natural RBM3- inductor; cooling.

The authors report that a single administration of ASO to exclude the poison exon, results in long-lasting increase of RBM3 expression in cells and mouse brains. In addition, in prion-diseased mice, this treatment leads to remarkable neuroprotection, with prevention of neuronal loss. The authors conclude that this novel approach used to stimulate the expression of RBM3 open the possibility to explore the beneficial effect of this cold-dependent protein to induce brain protection in acute brain injury or Alzheimer's disease, by avoiding the sides effect of hypothermia. This is a smart study, very well described, from an initial phase of ASO design, followed by an in vitro analysis and validation; and with a final in vivo testing. The authors have also a strong background the in the study of RBM3 that supports the results and the final conclusion of the analysis. Chemical agents or agonists to induce the pharmacological expression of RBM3 have been also suggested in other articles without good results so far, and this new approach is a smart alternative strategy to explore the therapeutic use of RBM3.

Some minor points related with the future application of ASO-RBM3 could be included in the discussion of the manuscript.

A recent study published in stroke (J Clin Med. 2022 Feb 11;11(4):949) and supported by previous studies (J. Cereb. Blood Flow Metab. 2019;39:2355-2367; Neuroscience. 2015;305:268-278.), suggests that the recombinant form of the hormone FGF21 could be used as an external therapy to modulate the expression of RBM3. This strategy seems more translational than ASO therapy.

We have included this approach and reference the 2019 study in the discussion on p7.

The use of ASO has been tested in experimental animals as a single intracerebroventricular injection that is no so invasive, but thinking in the potential application in old patients with Alzheimer or stroke (for instance), alternative and less invasive routes of administration are more convenient.

We agree, however, our study is a proof-of-principle of the approach and alternative delivery methods may become available. We do include a sentence highlighting the pursuit of alternative means of induction as future strategy on p7.

Based on the previous study published by same authors (Life Sci Alliance. 2021 Feb 9;4(4):e202000884); Was TrkB signalling analysed to guarantee that the protective effect was mediated by RBM3?

TrkB signalling is upstream of RBM3 splicing, initiated via BDNF binding of the receptor and induces RBM3 through a signalling cascade. This ASO is acting very much downstream of this, at the level of the pre-mRNA of RBM3 in the basal state (independent of activation of TrkB or other inducers) and we thus did not analyse TrkB signalling. We agree that it will be an interesting research direction to connect TrkB signalling with RBM3 AS-NMD in future research.

10th Feb 2023

Dear Florian,

Congratulations on a great revision! Overall, the referees have been positive. However, referee 2 has asked for a minor inclusion to the discussion that we would like you to include in an updated version.

When you submit your revised version, please also include a summary figure for the synopsis. The size should be 550 wide by 200-440 high (pixels). You can also use something from the figures if that is easier.

Please also include a "The paper explained" paragraph. EMBO Molecular Medicine articles are accompanied by a summary of the articles to emphasize the major findings in the paper and their medical implications for the non-specialist reader. Please provide a draft summary of your article highlighting: the medical issue you are addressing, the results obtained, and their clinical impact. This may be edited to ensure that readers understand the significance and context of your research. Please refer to any of our published articles for an example.

Thank you for the opportunity to consider your work for publication, I look forward to your revision.

Kind regards,

Kelly

Kelly M Anderson, PhD
Scientific Editor
EMBO Molecular Medicine

*** Instructions to submit your revised manuscript ***

*** PLEASE NOTE *** As part of the EMBO Publications transparent editorial process initiative (see our Editorial at https://www.embopress.org/doi/pdf/10.1002/emmm.201000094), EMBO Molecular Medicine will publish online a Review Process File to accompany accepted manuscripts.

In the event of acceptance, this file will be published in conjunction with your paper and will include the anonymous referee reports, your point-by-point response and all pertinent correspondence relating to the manuscript. If you do NOT want this file to be published, please inform the editorial office at contact@embomolmed.org.

When submitting your revised manuscript, please include:

1) a .docx formatted version of the manuscript text (including Figure legends and tables)

2) Separate figure files*

3) supplemental information as Expanded View and/or Appendix. Please carefully check the authors guidelines for formatting Expanded view and Appendix figures and tables at https://www.embopress.org/page/journal/17574684/authorguide#expandedview

4) a letter INCLUDING the reviewer's reports and your detailed responses to their comments (as Word file).

5) The paper explained: EMBO Molecular Medicine articles are accompanied by a summary of the articles to emphasize the major findings in the paper and their medical implications for the non-specialist reader. Please provide a draft summary of your article highlighting
- the medical issue you are addressing,
- the results obtained and
- their clinical impact.

This may be edited to ensure that readers understand the significance and context of the research.
Please refer to any of our published articles for an example.

6) For more information: There is space at the end of each article to list relevant web links for further consultation by our readers. Could you identify some relevant ones and provide such information as well? Some examples are patient associations, relevant databases, OMIM/proteins/genes links, author's websites, etc...

7) Author contributions: the contribution of every author must be detailed in a separate section.

8) EMBO Molecular Medicine now requires a complete author checklist (https://www.embopress.org/page/journal/17574684/authorguide) to be submitted with all revised manuscripts. Please use the checklist as guideline for the sort of information we need WITHIN the manuscript. The checklist should only be filled with page numbers were the information can be found. This is particularly important for animal reporting, antibody dilutions (missing) and exact values and n that should be indicted instead of a range.

9) Every published paper now includes a 'Synopsis' to further enhance discoverability. Synopses are displayed on the journal webpage and are freely accessible to all readers. They include a short stand first (maximum of 300 characters, including space) as well as 2-5 one sentence bullet points that summarise the paper. Please write the bullet points to summarise the key NEW findings. They should be designed to be complementary to the abstract - i.e. not repeat the same text. We encourage inclusion of key acronyms and quantitative information (maximum of 30 words / bullet point). Please use the passive voice. Please attach these in a separate file or send them by email, we will incorporate them accordingly.

You are also welcome to suggest a striking image or visual abstract to illustrate your article. If you do please provide a jpeg file 550 px-wide x 400-px high.

10) A Conflict of Interest statement should be provided in the main text

11) Please note that we now mandate that all corresponding authors list an ORCID digital identifier. This takes <90 seconds to complete. We encourage all authors to supply an ORCID identifier, which will be linked to their name for unambiguous name identification.

Currently, our records indicate that the ORCID for your account is 0000-0001-9377-9882.

Please click the link below to modify this ORCID:
Link Not Available

12) The system will prompt you to fill in your funding and payment information. This will allow Wiley to send you a quote for the article processing charge (APC) in case of acceptance. This quote takes into account any reduction or fee waivers that you may be eligible for. Authors do not need to pay any fees before their manuscript is accepted and transferred to our publisher.

*Additional important information regarding Figures

Each figure should be given in a separate file and should have the following resolution:
Graphs 800-1,200 DPI
Photos 400-800 DPI
Colour (only CMYK) 300-400 DPI"

Figures are not edited by the production team. All lettering should be the same size and style; figure panels should be indicated by capital letters (A, B, C etc). Gridlines are not allowed except for log plots. Figures should be numbered in the order of their appearance in the text with Arabic numerals. Each Figure must have a separate legend and a caption is needed for each panel.

*Additional important information regarding figures and illustrations can be found at https://bit.ly/EMBOPressFigurePreparationGuideline. See also figure legend preparation guidelines: https://www.embopress.org/page/journal/17574684/authorguide#figureformat

The system will prompt you to fill in your funding and payment information. This will allow Wiley to send you a quote for the article processing charge (APC) in case of acceptance. This quote takes into account any reduction or fee waivers that you may be eligible for. Authors do not need to pay any fees before their manuscript is accepted and transferred to our publisher.

***** Reviewer's comments *****

Referee #2 (Comments on Novelty/Model System for Author):

The RBM3 is a novel target that can be used for therapy in various neurological situations to protect neurons

Referee #2 (Remarks for Author):

The authors have addressed all the questions raised adequately. There is one point that was not addressed although it was given as a very general question so it is more specific here. This is simply a matter of pointing out the possibility that RBM3 has been studied in cancer. The increased expression of RBM3 has been shown to slow prostate cancer and plays a cancer promoting role in breast and colorectal cancer. This should be mentioned as this could be a problem for long term use but not in short term use like brain injury or cardiac surgery. This is relatively minor but should be mentioned.

Referee #3 (Comments on Novelty/Model System for Author):

The authors have completed properly the main comments and clarify the minor issues indicates in the first review.
I fully recommend this novel and interesting article for publication in EMBO Molecular Medicine journal

The authors addressed the remaining editorial issues.

18th Feb 2023

Dear Dr. Heyd,

Please find enclosed the final reports on your manuscript, I am pleased to inform you that your manuscript is accepted for publication in EMBO Molecular Medicine. Thank you for your comprehensive response to the referee concerns, it has been a pleasure to work with you to get this to the acceptance stage.

I will begin the final checks on your manuscript before submitting to the publisher next week. Once at the publisher, it will take about 3 weeks for your manuscript to be published online. As a reminder, the entire review process, including referee concerns and your point-by-point response, will be available to readers.

If you want to receive an e-mail alert regarding its publication as well as other EMBO Mol Med content, register here: embopress.org/alertsfeeds

I will be in touch throughout the final editorial process until publication. In the meantime, I hope you find time to celebrate!

Kind regards,

Kelly

Kelly M Anderson, PhD
Scientific Editor
EMBO Molecular Medicine
Follow us on Twitter @EmboMolMed

***** Reviewer's comments *****

*** *** *** IMPORTANT INFORMATION *** *** ***

Your article cannot be published until the publisher has received the appropriate signed license agreement. Once your article has been received by Wiley for production you will receive an email from Wiley's Author Services system, which will ask you to log in and will present you with the appropriate license for completion.

Should you be planning a Press Release on your article, please get in contact with embomolmed@wiley.com as early as possible, in order to coordinate publication and release dates.

PROOFS

You will receive the proofs by e-mail approximately 2 weeks after all relevant files have been sent o our Production Office. Please return them within 48 hours and if there should be any problems, please contact the production office at embopressproduction@wiley.com.

Please inform us if there is likely to be any difficulty in reaching you at the above address at that time. Failure to meet our deadlines may result in a delay of publication.

All further communications concerning your paper proofs should quote reference number EMM-2022-17157-V3 and be directed to the production office at embopressproduction@wiley.com.

Thank you,

Kelly M Anderson, PhD
Scientific Editor
EMBO Molecular Medicine

EMBO Press Author Checklist

Corresponding Author Name: Florian Heyd, Giovanna Mallucci and Marco Preussner
Journal Submitted to: EMBO Molecular Medicine
Manuscript Number: EMM-2022-17157

USEFUL LINKS FOR COMPLETING THIS FORM
The EMBO Journal - Author Guidelines
EMBO Reports - Author Guidelines
Molecular Systems Biology - Author Guidelines
EMBO Molecular Medicine - Author Guidelines

Reporting Checklist for Life Science Articles (updated January 2022)
This checklist is adapted from Materials Design Analysis Reporting (MDAR) Checklist for Authors. MDAR establishes a minimum set of requirements in transparent reporting in the life sciences (see Statement of Task: 10.31222/osf.io/9sm4x). Please follow the journal's guidelines in preparing your manuscript.
Please note that a copy of this checklist will be published alongside your article.

Abridged guidelines for figures
1. Data
The data shown in figures should satisfy the following conditions:
- the data were obtained and processed according to the field's best practice and are presented to reflect the results of the experiments in an accurate and unbiased manner.
- ideally, figure panels should include only measurements that are directly comparable to each other and obtained with the same assay.
- plots include clearly labeled error bars for independent experiments and sample sizes. Unless justified, error bars should not be shown for technical replicates.
- if n<5, the individual data points from each experiment should be plotted. Any statistical test employed should be justified.
- Source Data should be included to report the data underlying figures according to the guidelines set out in the authorship guidelines on Data Presentation.

2. Captions
Each figure caption should contain the following information, for each panel where they are relevant:
- a specification of the experimental system investigated (eg cell line, species name).
- the assay(s) and method(s) used to carry out the reported observations and measurements.
- an explicit mention of the biological and chemical entity(ies) that are being measured.
- an explicit mention of the biological and chemical entity(ies) that are altered/varied/perturbed in a controlled manner.
- the exact sample size (n) for each experimental group/condition, given as a number, not a range;
- a description of the sample collection allowing the reader to understand whether the samples represent technical or biological replicates (including how many animals, litters, cultures, etc.).
- a statement of how many times the experiment shown was independently replicated in the laboratory.
- definitions of statistical methods and measures:

- common tests, such as t-test (please specify whether paired vs. unpaired), simple χ2 tests, Wilcoxon and Mann-Whitney tests, can be unambiguously identified by name only, but more complex techniques should be described in the methods section;

- are tests one-sided or two-sided?
- are there adjustments for multiple comparisons?
- exact statistical test results, e.g., P values = x but not P values < x;
- definition of 'center values' as median or average;
- definition of error bars as s.d. or s.e.m.

Please complete ALL of the questions below.
Select "Not Applicable" only when the requested information is not relevant for your study.

Materials

Newly Created Materials	Information included in the manuscript?	In which section is the information available? (Reagents and Tools Table, Materials and Methods, Figures, Data Availability Section)
New materials and reagents need to be available; do any restrictions apply?	Not Applicable	

Antibodies	Information included in the manuscript?	In which section is the information available? (Reagents and Tools Table, Materials and Methods, Figures, Data Availability Section)
For **antibodies** provide the following information:<br>- Commercial antibodies: RRID (if possible) or supplier name, catalogue number and or/clone number<br>- Non-commercial: RRID or citation	Yes	

DNA and RNA sequences	Information included in the manuscript?	In which section is the information available? (Reagents and Tools Table, Materials and Methods, Figures, Data Availability Section)
**Short novel DNA or RNA including primers, probes**: provide the sequences.	Yes	Expanded View Table and Materials and Methods

Cell materials	Information included in the manuscript?	In which section is the information available? (Reagents and Tools Table, Materials and Methods, Figures, Data Availability Section)
**Cell lines:** Provide species information, strain. Provide accession number in repository **OR** supplier name, catalog number, clone number, and/**OR** RRID.	Yes	Materials and Methods
**Primary cultures:** Provide species, strain, sex of origin, genetic modification status.	Yes	Materials and Methods
Report if the cell lines were recently **authenticated** (e.g., by STR profiling) and tested for mycoplasma contamination.	Yes	Materials and Methods

Experimental animals	Information included in the manuscript?	In which section is the information available? (Reagents and Tools Table, Materials and Methods, Figures, Data Availability Section)
**Laboratory animals or Model organisms:** Provide species, strain, sex, age, genetic modification status. Provide accession number in repository **OR** supplier name, catalog number, clone number, **OR** RRID.	Yes	materials and methods and figures and text
**Animal observed in or captured from the field:** Provide species, sex, and age where possible.	Not Applicable	
Please detail **housing and husbandry conditions**.	Yes	materials and methods

Plants and microbes	Information included in the manuscript?	In which section is the information available? (Reagents and Tools Table, Materials and Methods, Figures, Data Availability Section)

**Plants:** provide species and strain, ecotype and cultivar where relevant, unique accession number if available, and source (including location for collected wild specimens).	Not Applicable	
**Microbes:** provide species and strain, unique accession number if available, and source.	Not Applicable	

Human research participants	Information included in the manuscript?	In which section is the information available? (Reagents and Tools Table, Materials and Methods, Figures, Data Availability Section)
If collected and within the bounds of privacy constraints report on age, sex and gender or ethnicity for all study participants.	Not Applicable	
**Core facilities**	**Information included in the manuscript?**	**In which section is the information available?** (Reagents and Tools Table, Materials and Methods, Figures, Data Availability Section)
If your work benefited from core facilities, was their service mentioned in the acknowledgments section?	Not Applicable	

Design

Study protocol	Information included in the manuscript?	In which section is the information available? (Reagents and Tools Table, Materials and Methods, Figures, Data Availability Section)
If study protocol has been **pre-registered, provide DOI in the manuscript**. For clinical trials, provide the trial registration number **OR** cite DOI.	Not Applicable	
Report the **clinical trial registration number** (at ClinicalTrials.gov or equivalent), where applicable.	Not Applicable	

Laboratory protocol	Information included in the manuscript?	In which section is the information available? (Reagents and Tools Table, Materials and Methods, Figures, Data Availability Section)
Provide DOI OR other citation details if **external detailed step-by-step protocols** are available.	Not Applicable	

Experimental study design and statistics	Information included in the manuscript?	In which section is the information available? (Reagents and Tools Table, Materials and Methods, Figures, Data Availability Section)
Include a statement about **sample size** estimate even if no statistical methods were used.	Yes	Materials and Methods, Figure Legends
Were any steps taken to minimize the effects of subjective bias when allocating animals/samples to treatment (e.g. **randomization procedure**)? If yes, have they been described?	Yes	Materials and Methods
Include a statement about **blinding** even if no blinding was done.	Yes	Materials and Methods
Describe **inclusion/exclusion criteria** if samples or animals were excluded from the analysis. Were the criteria pre-established?<br><br>If sample or data points were omitted from analysis, report if this was due to attrition or intentional exclusion and provide justification.	Not Applicable	
For every figure, are **statistical tests** justified as appropriate? Do the data meet the assumptions of the tests (e.g., normal distribution)? Describe any methods used to assess it. Is there an estimate of variation within each group of data? Is the variance similar between the groups that are being statistically compared?	Yes	Materials and Methods, Figure Legends

Sample definition and in-laboratory replication	Information included in the manuscript?	In which section is the information available? (Reagents and Tools Table, Materials and Methods, Figures, Data Availability Section)
In the figure legends: state number of times the experiment was **replicated** in laboratory.	Yes	figure legend
In the figure legends: define whether data describe **technical or biological replicates**.	Yes	Materials and Methods

Ethics

Ethics	Information included in the manuscript?	In which section is the information available? (Reagents and Tools Table, Materials and Methods, Figures, Data Availability Section)
Studies involving **human participants**: State details of **authority granting ethics approval** (IRB or equivalent committee(s), provide reference number for approval.	Not Applicable	
Studies involving **human participants**: Include a statement confirming that **informed consent** was obtained from all subjects and that the experiments conformed to the principles set out in the WMA Declaration of Helsinki and the Department of Health and Human Services Belmont Report.	Not Applicable	
Studies involving **human participants:** For publication of **patient photos**, include a statement confirming that consent to publish was obtained.	Not Applicable	
Studies involving experimental **animals**: State details of **authority granting ethics approva**l (IRB or equivalent committee(s), provide reference number for approval. Include a statement of compliance with ethical regulations.	Yes	Materials and Methods
Studies involving **specimen and field samples:** State if relevant **permits** obtained, provide details of authority approving study; if none were required, explain why.	Not Applicable	

Dual Use Research of Concern (DURC)	Information included in the manuscript?	In which section is the information available? (Reagents and Tools Table, Materials and Methods, Figures, Data Availability Section)
Could your study fall under dual use research restrictions? Please check biosecurity documents and list of **select agents and toxins** (CDC): https://www.selectagents.gov/sat/list.htm	Not Applicable	
If you used a select agent, is the security level of the lab appropriate and reported in the manuscript?	Not Applicable	

If a study is subject to dual use research of concern regulations, is the name of the **authority granting approval and reference number** for the regulatory approval provided in the manuscript?	Not Applicable	

Reporting

The MDAR framework recommends adoption of discipline-specific guidelines, established and endorsed through community initiatives. Journals have their own policy about requiring specific guidelines and recommendations to complement MDAR.

Adherence to community standards	Information included in the manuscript?	In which section is the information available? (Reagents and Tools Table, Materials and Methods, Figures, Data Availability Section)
State if relevant guidelines or checklists (e.g., **ICMJE, MIBBI, ARRIVE, PRISMA**) have been followed or provided.	Yes	Materials and Methods
For **tumor marker prognostic studies,** we recommend that you follow the **REMARK** reporting guidelines (see link list at top right). See author guidelines, under 'Reporting Guidelines'. Please confirm you have followed these guidelines.	Not Applicable	
For **phase II and III randomized controlled trials**, please refer to the **CONSORT** flow diagram (see link list at top right) and submit the CONSORT checklist (see link list at top right) with your submission. See author guidelines, under 'Reporting Guidelines'. Please confirm you have submitted this list.	Not Applicable	

Data Availability

Data availability	Information included in the manuscript?	In which section is the information available? (Reagents and Tools Table, Materials and Methods, Figures, Data Availability Section)
Have **primary datasets** been deposited according to the journal's guidelines (see 'Data Deposition' section) and the respective accession numbers provided in the Data Availability Section?	Not Applicable	
Were **human clinical and genomic datasets** deposited in a public access-controlled repository in accordance to ethical obligations to the patients and to the applicable consent agreement?	Not Applicable	
Are **computational models** that are central and integral to a study available without restrictions in a machine-readable form? Were the relevant accession numbers or links provided?	Not Applicable	
If publicly available data were reused, provide the respective **data citations in the reference list**.	Yes	Materials and Methods, Figure Legends
